# Elucidating the role of EPPK1 in lung adenocarcinoma development

**DOI:** 10.1186/s12885-024-12185-x

**Published:** 2024-04-10

**Authors:** Ken Arimura, Michael Kammer, S. M. Jamshedur Rahman, Chen Sheau-Chiann, Shilin Zhao, Chen Heidi, Rosana Eisenberg, Yong Zou, Sanja Antic, Bradley Richmond, Etsuko Tagaya, Eric Grogan, Pierre Massion, Fabien Maldonado

**Affiliations:** 1 Division of Allergy, Pulmonary and Critical Care Medicine, Vanderbilt University Medical Center, Nashville, TN USA; 2 Department of Respiratory Medicine, Tokyo Women’s Medical University, Tokyo, Japan; 3 Department of Biostatistics, Vanderbilt University Medical Center, Nashville, TN USA; 4 Department of Pathology, Microbiology, and Immunology, Vanderbilt University Medical Center, Nashville, TN USA; 5 Department of Thoracic Surgery, Vanderbilt University Medical Center, Nashville, TN USA

**Keywords:** CRISPR-Cas9, Epiplakin 1, Epithelial-to-mesenchymal transition, MYC/p53 pathway

## Abstract

**Background:**

We recently found that epiplakin 1 (EPPK1) alterations were present in 12% of lung adenocarcinoma (LUAD) cases and were associated with a poor prognosis in early-stage LUAD when combined with other molecular alterations. This study aimed to identify a probable crucial role for EPPK1 in cancer development.

**Methods:**

EPPK1 mRNA and protein expression was analyzed with clinical variables. Normal bronchial epithelial cell lines were exposed to cigarette smoke for 16 weeks to determine whether EPPK1 protein expression was altered after exposure. Further, we used CRISPR-Cas9 to knock out (KO) EPPK1 in LUAD cell lines and observed how the cancer cells were altered functionally and genetically.

**Results:**

EPPK1 protein expression was associated with smoking and poor prognosis in early-stage LUAD. Moreover, a consequential mesenchymal-to-epithelial transition was observed, subsequently resulting in diminished cell proliferation and invasion after EPPK1 KO. RNA sequencing revealed that EPPK1 KO induced downregulation of 11 oncogenes, 75 anti-apoptosis, and 22 angiogenesis genes while upregulating 8 tumor suppressors and 12 anti-cell growth genes. We also observed the downregulation of MYC and upregulation of p53 expression at both protein and RNA levels following EPPK1 KO. Gene ontology enrichment analysis of molecular functions highlighted the correlation of EPPK1 with the regulation of mesenchymal cell proliferation, mesenchymal differentiation, angiogenesis, and cell growth after EPPK1 KO.

**Conclusions:**

Our data suggest that EPPK1 is linked to smoking, epithelial to mesenchymal transition, and the regulation of cancer progression, indicating its potential as a therapeutic target for LUAD.

**Supplementary Information:**

The online version contains supplementary material available at 10.1186/s12885-024-12185-x.

## Background

Lung cancer is a leading cause of cancer-related mortality worldwide. Current treatments for lung adenocarcinoma (LUAD) primarily target specific molecular alterations, including epidermal growth factor receptor alterations, anaplastic lymphoma kinase fusions, ROS1 proto-oncogene receptor tyrosine kinase rearrangements, transfection fusion-induced rearrangements, B-Raf proto-oncogene serine/threonine kinase alterations, and Kirsten rat sarcoma viral oncogene homolog alterations [1–6]. These treatments have demonstrated excellent response rates in some patients [1–6]. However, despite recent therapeutic advances, many tumors eventually develop resistance to chemotherapy, leading to disease progression or recurrence. The underlying reasons for chemotherapy resistance are diverse and not yet fully understood [7]. Therefore, continued research efforts focused on identifying novel molecular targets and understanding the mechanisms of resistance and disease progression are crucial for improving the overall survival (OS) of patients with lung cancer. One potential target of interest is epiplakin 1 (EPPK1), which is located on chromosome 8q24.3 and encodes a protein belonging to the plakin family. EPPK1, with a molecular mass of 450 kDa, plays a role in linking intermediate filaments and regulating their reorganization in response to stress [8–10]. In a recent study, we observed alterations in EPPK1 in 12% of patients with early-stage LUAD [11]. When combined with alterations in other genes, such as phosphatidylinositol-4,5-bisphosphate 3-kinase catalytic subunit gamma (PIK3CG), ataxia telangiectasia mutated (ATM), E1A binding protein P300 (EP300), and lysine methyltransferase 2C (KMT2C), EPPK1 gene alterations were associated with a poor prognosis in early-stage LUAD [11]. Additionally, prior studies have suggested that downregulation or absence of EPPK1 promotes cell migration and proliferation in the human corneal epithelium [9, 12]. While the exact role of EPPK1 remains unclear, our previous findings led us to hypothesize that EPPK1 plays a crucial role in cancer development. Therefore, this study aimed to determine the expression and alterations of EPPK1 in various cancer types, evaluate its association with clinical outcomes in non-small cell lung cancer (NSCLC), and elucidate the functional role of EPPK1 and the genomic alterations that occur following EPPK1 knockout (KO) in lung cancer cells.

## Methods

### Tissue microarrays (TMAs)

TMAs were prepared from the surgical specimens obtained from the Vanderbilt-Ingram Cancer Center Thoracic Biorepository. This biorepository houses tissue samples, along with sex as a biological variable, age, and clinical data from the Vanderbilt University Medical Center (VUMC) and the associated Veterans Affairs Hospital, Tennessee Valley Healthcare System. The TMA dataset comprised 35 cases for normal lung tissues and 295 patients diagnosed with NSCLC, including 140 patients with LUAD and 155 patients with lung squamous cell carcinoma (LUSC). The study received approval from the VUMC Institutional Review Board (IRB approval number: 000616), and all patients provided informed consent before their tumor specimens were collected. The study design did not involve randomization or blinding trials.

### Cell culture

Lung cancer cell lines (A549, H2009, H1819, Calu3, H23, H1993, H226, H460, and H1299) and the normal bronchial epithelial cell line (BEAS2B) were obtained from the American Type Culture Collection, as previously described [7]. The cell lines were cultured in Dulbecco’s modified Eagle’s medium (DMEM) (Thermo Fisher Scientific, Waltham, MA, USA) or RPMI-1640 medium (Thermo Fisher Scientific), supplemented with 1% penicillin–streptomycin and 10% fetal bovine serum.

### Immunohistochemistry (IHC)

The expression of EPPK1 was assessed in the lung cancer cell line TMAs, normal lung TMAs, and normal bronchial epithelial cell line microarray. The TMAs were stained using antibodies for EPPK1 (#2–49597, rabbit polyclonal, 1:1000; NOVUS, Colorado, USA) and programmed death-ligand 1 (PD-L1) (#228415, clone 73–10, rabbit monoclonal, 1:500; Abcam, Cambridge, UK). The IHC score for EPPK1 was defined as cytoplasmic staining by multiplying the staining distribution scores of 0 (0%), 0.1 (1–9%), 0.5 (10–49%), 1 (50–100%) by the staining intensity score (0–3) [7]. The patients were classified into two equal groups based on the median score, indicating high and low EPPK1 expression. The IHC score for PD-L1 was defined as membrane staining present in at least 1% of cells, regardless of the staining distribution and intensity [13, 14]. The expression levels of EPPK1 and PD-L1 were evaluated by an experienced pathologist.

### The Cancer Genome Atlas and TIMER analysis

We investigated the EPPK1 gene alterations and mRNA expression in 36 different human cancer types using The Cancer Genome Atlas (TCGA) pan-cancer cohort, which encompasses a wide range of human cancer types. The objective was to examine their association with OS (UCSC Xena, RRID:SCR_018938) [15]. Moreover, this cohort allowed us to compare mRNA expression levels between normal and tumor tissues across 17 cancer types. For visualization and analysis, we used TIMER (RRID:SCR_018737), a tool specifically designed for analyzing data from TCGA [16]. To facilitate the evaluation of mRNA expression differences, we converted the expression values to the log2 of transcripts per million and assessed the variation between tumor tissues and normal tissues adjacent to tumor tissues.

### Western blotting (WB)

Cell protein lysates were extracted, and WB analysis was conducted using a standard protocol [17]. The bands were visualized using specific antibodies against the following proteins: EPPK1 (#1488502, 1:1000, rabbit polyclonal, BioSource, Camarillo, California, USA), γH2AX (#97185, 1:1000, rabbit monoclonal, Cell Signaling Technology, Massachusetts, USA), E-cadherin (#610182, 1:1000, mouse monoclonal, BD Transduction Laboratories, California, USA), Vimentin (#5741, 1:1000, rabbit monoclonal, Cell Signaling Technology), c-MYC (#5605, 1:1000, D84C12, rabbit monoclonal, Cell Signaling Technology), p53 (#2527, 1:1000, 7F5, rabbit monoclonal, Cell Signaling Technology), and β-actin (#2228, 1:5000, mouse monoclonal, Sigma, Missouri, USA).

### Cigarette smoking exposure

A total of 5 × 10^5^ BEAS2B control cells and BEAS2B cells exposed to smoking were seeded in a transwell with a 0.4 µm pore size and 500 µL of DMEM. Simultaneously, a 6-well plate (#3450, Corning Life Sciences, Massachusetts, USA) was filled with 1500 µL of DMEM, and the cells were cultured for 3 days before cigarette smoke exposure. Prior to smoke exposure, the medium in the transwell for both control and smoke-exposed cells was aspirated. Smoke-exposed cells were then placed in a smoking machine (Smoke Inhalation Unit 24, Promech Lab, Scania, Sweden), while control cells were kept in ambient conditions in an outer incubator during the smoking exposure period. The cells were exposed to cigarette smoke from three research cigarettes (3R4F; Cooperation Centre for Scientific Research Relative to Tobacco, Kentucky, USA) each weekday for four months. Following the cigarette smoke exposure, 500 µL of the medium was returned to each transwell. Weekly, each cell line was harvested, and the cells were seeded in a transwell again. The smoking density was measured using a Microdust Pro 880 nm Aerosol Monitoring device (Casella, New York, USA), and carbon monoxide levels in the smoking chamber were measured using the Auto Calibrating Carbon Monoxide detector (#UTLC11, UEi, California, USA). All experiments were performed in triplicate and repeated three times.

### Immunofluorescence

The cells were seeded in a chamber slide (Nunc Lab-Tek II Chamber Slide system, NUNC) and incubated overnight in a cell incubator. Subsequently, the cells were fixed with 4% formalin and stained with a mounting medium containing DAPI (#H1200, Vectashield mounting medium with DAPI, Vector Laboratories, California, USA) to visualize the nuclei. Immunostaining was performed using an antibody for γH2AX (#97185, 1:400, rabbit monoclonal, Cell Signaling Technology). After staining, the cells were examined in five imaging fields using a fluorescence microscope with 20 × magnification (IX51 Inverted Microscope, Olympus, Massachusetts, USA). The fluorescence intensity was measured, and the analysis was conducted using cell image analysis software (CellProfiler Image Analysis Software, RRID:SCR_007358). Each experiment was performed in triplicate.

### Cell invasion assays

Transwell inserts with an 8.0 µm pore size, placed in a 24-well plate (#3422, Corning Life Sciences), were coated with Matrigel (#356234, Corning Life Sciences) to create a basement membrane on both the upper and lower surfaces of the transwell membranes. A total of 5 × 10^5^ cells were seeded in the upper chamber of the transwell with 100 µL of DMEM, while the wells of the 24-well plate were filled with 500 µL of DMEM. After incubating the cells for 24 h, the cells on the upper surface of the transwell membrane were gently scraped off, and the cells on the lower surface of the membrane were fixed with 4% formalin and stained with a mounting medium containing DAPI. Cell images were captured in five fields of view using a fluorescence microscope with a 20x magnification, and the number of cells was quantified using cell image analysis software (ImageJ, RRID:SCR_003070). Each experiment was performed in triplicate.

### Cell proliferation assays

Cells were seeded at a density of 1 × 10^3^ cells per well in 96-well plates (Thermo Fisher Scientific, #167008, Nunc MicroWell 96-Well Microplates). The cells were then cultured for 2, 4, and 6 days. To measure cell counts, the CyQUANT assay (Thermo Fisher Scientific, #C7026, CyQUANT Cell Proliferation Assay) was used, and the fluorescence was measured using a plate reader. Each experiment was independently repeated three times, with six wells analyzed per experiment.

### RNA sequencing

RNA was extracted using the RNeasy Plus Mini Kit (#74034; Qiagen, Redwood City, CA, USA). The RNA sequencing libraries contained 300 ng of RNA and were prepared using the NEBNext Ultra II Directional RNA Library Prep Kit (#E7760L, New England Bio Labs, County Road, MA, USA). Fragmentation, cDNA synthesis, end-repair/dA-tailing, adaptor ligation, and PCR enrichment were performed according to the manufacturer’s instructions. The quality of individual libraries was assessed using an Agilent 2100 Bioanalyzer (Agilent, Santa Clara, CA, USA), and quantification was performed using a Qubit Fluorometer (Thermo Fisher Scientific). The adapter-ligated material was estimated using qPCR before normalization and pooling for sequencing. The libraries were sequenced on a NovaSeq 6000 platform using 150 bp paired-end reads. Base calling was performed using RTA (Illumina, San Diego, CA, USA), and data quality control was conducted using the Vanderbilt Technologies for Advanced Genomics core facility. Poor-quality reads were trimmed, and adapter sequences were removed. The reads were then aligned to the human genome using STAR (STAR, RRID:SCR_004463) [18] and quantified using featureCounts [19]. Poor-quality RNA-Seq experiments were excluded from the analysis. Differential gene expression analysis was performed using the R package DESeq2 (DESeq, RRID:SCR_000154) [20]. A heat map was generated, and gene ontology (GO) enrichment analysis with a false discovery ratio was conducted using the R package to visualize gene expression across the samples. All experiments were performed in triplicate.

### Statistical analyses

This is a retrospective observational study. The data were analyzed using R software (version 4.1.2; The R Foundation for Statistical Computing, Vienna, Austria). Descriptive statistics, including median and interquartile range for continuous variables, and frequency and percentage for categorical variables, were employed to summarize the clinical data. Wilcoxon rank-sum test was used for two-group comparisons of continuous variables in both TCGA and VUMC clinical data. The association between categorical variables was assessed using Fisher’s exact test or Pearson’s chi-squared test. Two-way ANOVA was used to examine whether there was an interaction between treatment groups and time for immunofluorescence (nuclei intensity) and cell proliferation (fluorescence intensity) data. Based on model-based (least-squares) means, the Wald test was employed to estimate and compare the treatment group means at the selected times. A 95% confidence interval (CI) for the mean difference was also reported. Standard residual analysis was conducted, and Bonferroni-adjusted *p*-values were used for multiple comparisons.

The OS of patients in the high and low EPPK1 expression groups was estimated using the Kaplan–Meier (KM) method and compared using the log-rank test. To determine the effect of high and low EPPK1 levels on death over time, the hazard ratio (HR) with a 95% CI was calculated using a univariate Cox regression model. A multivariable Cox regression model, adjusted for clinical variables (age, gender, smoking status, and pack-year), was used to investigate the association between OS and the high/low EPPK1 groups. The multivariate model included an interaction term between EPPK1 and the cancer stage. Subgroup analyses were performed for the early and late stages. In the multivariate Cox regression model for the early stages, adjustment was made only for gender, which was considered the primary confounder. The proportional hazards assumption was evaluated using the chi-square goodness-of-fit test and graphical diagnostics based on the scaled Schoenfeld residuals. Statistical significance was set at *P* < 0.05.

## Results

### EPPK1 is associated with poor prognosis in multiple cancers, and EPPK1 mRNA is overexpressed in LUAD and LUSC in TCGA

**Fig. 1:**
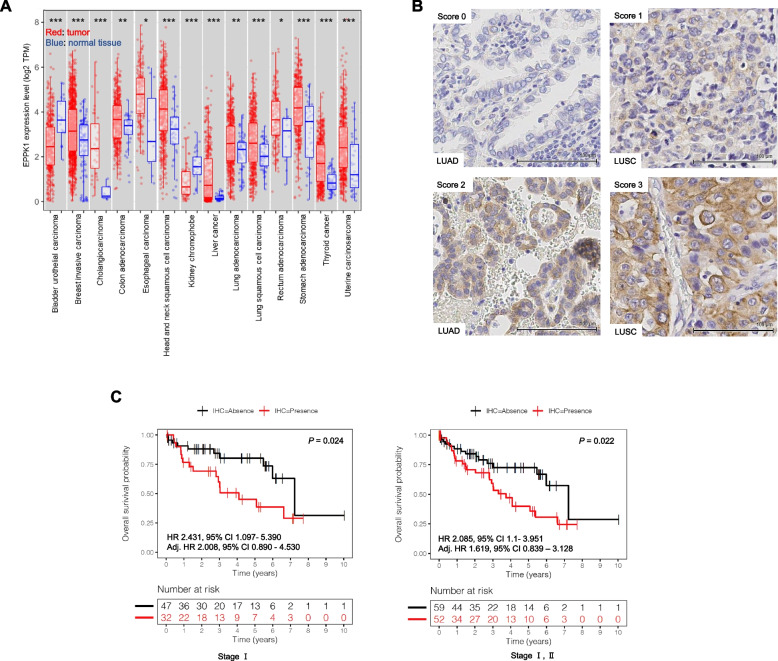
Analysis of EPPK1 mRNA and protein expression levels in multiple cancers. **A** Comparison of EPPK1 mRNA levels between tumor and normal tissues in various human cancers using the Wilcoxson rank sum test. EPPK1 mRNA was overexpressed in breast invasive carcinoma, cholangiocarcinoma, colon adenocarcinoma, esophageal carcinoma, head and neck squamous cell carcinoma, kidney chromophobe, liver cancer, lung adenocarcinoma, lung squamous cell carcinoma, rectum adenocarcinoma, stomach adenocarcinoma, thyroid cancer, uterine carcinosarcoma compared to levels in their respective normal tissues. In contrast, EPPK1 mRNA was overexpressed in normal tissue adjacent to bladder urothelial carcinoma and kidney chromophobe tissue compared to levels in their respective tumor tissues. Red: tumor, Blue: normal tissue, ***P < 0.001, **P < 0.01, *P < 0.05. **B** Representative image of EPPK1 protein expression in lung adenocarcinoma (LUAD) and lung squamous cell carcinoma (LUSC) (20x). **C** Overall survival analysis using Kaplan–Meier for EPPK1 protein expression in early-stage LUAD. Log-rank test demonstrates that elevated EPPK1 protein expression correlates with poor prognosis (*P* = 0.024 in stage I, *P* = 0.022 in stage I + II). Hazard ratio (HR) with the 95% confidence interval (CI) is reported using the univariate Cox regression model. Adjusted (Adj.) HR is presented using a multivariable Cox regression model adjusted for gender

To investigate the potential association of EPPK1 with OS across multiple cancers, we analyzed mRNA expression data from TCGA database. Our findings revealed a significant correlation between EPPK1 expression and poor prognosis in various cancers (see Table 1). These results indicate that EPPK1 may serve as a promising prognostic biomarker for these malignancies. Furthermore, we examined the mRNA expression of EPPK1 to assess its relationship with tumor development. By comparing EPPK1 mRNA expression levels between normal and tumor tissues from multiple cancer types in TCGA dataset, we made several noteworthy observations. Firstly, we observed elevated expression of EPPK1 mRNA in LUAD (*P* < 0.01, see Fig. 1A) and LUSC (*P* < 0.001, see Fig. 1A) as well as in several other cancers, in comparison to that in normal tissues (see Fig. 1A). These findings highlight the potential significance of EPPK1 in progression across different cancer types.

**Table 1:** Association of EPPK1 with poor prognosis of multiple cancers in TCGA

Cancer type	Outcome	N	HR	95% CI	*P* value
Lower grade glioma	OS	514	1.851	1.013–3.383	0.045
Mesothelioma	OS	86	1.211	1.009–1.454	0.04
Melanoma	OS	462	1.364	1.138–1.637	0.001
Pancreatic adenocarcinoma	OS	179	1.24	1.027–1.498	0.025
Uterine carcinoma	OS	544	1.283	1.107–1.487	0.001

*N* Number of patients, *OS* Overall survival, *HR* hazard ratio, *CI* confidence interval

### EPPK1 correlates with poor prognosis in early-stage LUAD

To evaluate EPPK1 as a potential biomarker, we analyzed its protein expression in 280 patients with NSCLC, including 140 with LUAD and 140 with LUSC. We examined the association among EPPK1 protein expression, clinical variables, and OS. Patient characteristics, such as age, gender, smoking history, histology, pathological stage, and chronic obstructive pulmonary disease status, were summarized by histology and EPPK1 protein expression (Tables 2, 3 and 4). EPPK1 protein was found to be expressed in the cytoplasm of resected lung cancer tissue (Fig. 1B) and lung cancer cell lines (Fig. S1). However, EPPK1 was not expressed in resected normal lung tissue or normal bronchial epithelial cell line microarrays (Fig. S2). We did not observe any relevant interaction effect between EPPK1 and PD-L1 status (Fig. S3) or gene alterations. However, in patients with LUAD, the interaction effect between EPPK1 and stage (early vs. late stage) was associated with OS in the multivariable Cox regression model adjusted for gender and pack-years (*P* < 0.01, LUAD in Table 5). This indicates that the impact of EPPK1 on OS differed between the early and late stages. In the early stages, there was a significant difference in survival between the EPPK1-high and -low groups (Fig. 1C, log-rank test, *P* = 0.024 in stage I; *P* = 0.022 in stages I and II; Fig. 1C). Patients in the EPPK1-high group had a 2.431 times higher likelihood of death than those in the low EPPK1 expression group in stage I (HR with CI = 2.431 [1.097–5.39], Wald test: *P* = 0.03, Fig. 1C), and a 2.085 times higher likelihood in stages I and II (HR with CI = 2.085 [1.1–3.951], Wald test: *P* = 0.02 in stages I and II, Fig. 1C). After adjusting for gender in the multivariable analysis, the risk of death in the high-expression EPPK1 group was reduced to 2.008 times higher than that in the low-expression EPPK1 group in stage I (adjusted [adj.] HR with CI = 2.008 [0.890–4.530], Wald test: *P* = 0.09 in stage I) and 2.619 times higher in stages I and II (adj. HR with CI = 1.619 [0.839–3.128], Wald test: *P* = 0.15 in stages I and II). In late-stage LUAD, there was no difference in OS between the EPPK1-high and -low groups after adjusting for gender and pack-years using multivariable Cox regression analysis. Additionally, there was no difference in OS when considering the interaction effect between EPPK1 and stage or the main effect of EPPK1 in the LUSC group (adj. *P* = 0.96, LUSC in Table 5). These findings suggest that EPPK1 expression plays a critical role in cancer development in LUAD and could serve as a prognostic biomarker for early-stage LUAD.

**Table 2:** Characteristics of patients with NSCLC

Characteristic	N	All, *N* = 295
Age	294	66 (60–72)
Gender	294	
Female		121 (41.2%)
Male		173 (58.8%)
Smoking history	292	
Ever		267 (91.4%)
Never		25 (8.6%)
Histology	295	
LUAD		140 (47.5%)
LUSC		155 (52.5%)
Stage	295	
I		114 (38.6%)
II		44 (14.9%)
III		93 (31.5%)
IV		44 (14.9%)
COPD	237	96 (40.5%)
EPPK1	280	140 (50.0%)
Low		140 (50.0%)
High		140 (50.0%)
Median (25%-75%); n (%)		

*NSCLC* Non-small cell lung cancer, *N* Number of patients, *LUAD* Lung adenocarcinoma, *LUSC* Lung squamous cell carcinoma, *COPD* Chronic obstructive pulmonary disease

**Table 3:** Patient characteristics by EPPK1 expression levels in LUAD

Characteristic	N	All LUAD^1^ (*N* = 140)	EPPK1 Low^1^ (*N* = 72)	EPPK1 High^1^ (*N* = 68)	*P* value^2^
Age	140	67 (60–71.25)	65 (59–71.25)	67 (61–71.25)	0.48
Gender	140				0.02
Female		78 (55.7%)	47 (65.3%)	31 (45.6%)	
Male		62 (44.3%)	25 (34.7%)	37 (54.4%)	
Smoking history	140				0.15
Ever		117 (83.6%)	57 (79.2%)	60 (88.2%)	
Never		23 (16.4%)	15 (20.8%)	8 (11.8%)	
Pack years	136	40 (12.75–60)	29 (10–55)	45 (20–61)	0.03
Performance Status	136	15 (11.0%)	6 (8.7%)	9 (13.4%)	0.38
Stage	140				0.13
I		79 (56.4%)	47 (65.3%)	32 (47.1%)	
II		32 (22.9%)	12 (16.7%)	20 (29.4%)	
III		24 (17.1%)	10 (13.9%)	14 (20.6%)	
IV		5 (3.6%)	3 (4.2%)	2 (2.9%)	
COPD	131	38 (29.0%)	14 (20.6%)	24 (38.1%)	0.03
Gene alterations	71				
KRAS		47 (33.6%)	24 (33.3%)	23 (33.8%)	0.95
EGFR		17 (12.1%)	12 (16.7%)	5 (7.4%)	0.09
BRAF		3 (2.1%)	2 (2.8%)	1 (1.5%)	0.99
PIK3CA		2 (1.4%)	1 (1.4%)	1 (1.5%)	0.99
MEK		1 (0.7%)	0 (0.0%)	1 (1.5%)	0.49
ALK		1 (0.7%)	1 (1.4%)	0 (0.0%)	0.99

*LUAD* Lung adenocarcinoma, *N* Number of patients, *COPD* Chronic obstructive pulmonary disease

^1^Median (25%-75%); n (%)

^2^Wilcoxon rank sum test; Pearson’s Chi-squared test; Fisher’s exact test

**Table 4:** Patient characteristics by EPPK1 expression levels in LUSC

Characteristic	N	All LUSC^1^ (*N* = 140)	EPPK1 Low^1^ (*N* = 68)	EPPK1 High^1^ (*N* = 72)	*P* value^2^
Age	139	66 (60–72)	66 (60–73)	64.5 (59.75–70)	0.26
Gender	139				0.66
Female		35 (25.2%)	18 (26.9%)	17 (23.6%)	
Male		104 (74.8%)	49 (73.1%)	55 (76.4%)	
Smoking Status	137				0.23
Ever		135 (98.5%)	64 (97.0%)	71 (100.0%)	
Never		2 (1.5%)	2 (3.0%)	0 (0.0%)	
Pack Year	139	50 (40–72.5)	40 (27.5–60)	60 (40–80)	< 0.001
Performance Status	72				0.47
0		24 (33.3%)	12 (33.3%)	12 (33.3%)	
1		21 (29.2%)	8 (22.2%)	13 (36.1%)	
2		8 (11.1%)	6 (16.7%)	2 (5.6%)	
3		12 (16.7%)	7 (19.4%)	5 (13.9%)	
4		7 (9.7%)	3 (8.3%)	4 (11.1%)	
Stage	140				0.43
I		29 (20.7%)	13 (19.1%)	16 (22.2%)	
II		12 (8.6%)	4 (5.9%)	8 (11.1%)	
III		65 (46.4%)	36 (52.9%)	29 (40.3%)	
IV		34 (24.3%)	15 (22.1%)	19 (26.4%)	
COPD	97	54 (55.7%)	25 (53.2%)	29 (58.0%)	0.63

*LUSC* Lung squamous cell carcinoma, *N* Number of patients, *COPD* Chronic obstructive pulmonary disease

^1^Median (25%-75%); n (%)

^2^Wilcoxon rank sum test; Pearson’s Chi-squared test; Fisher’s exact test

**Table 5:** Interaction effect of EPPK1 and stage in a multivariable Cox regression model adjusted for gender and pack year for each histology

Histology	Variable	Coef	exp(Coef)	se(Coef)	z	*P* value
LUAD	Pack Years	0.01	1.01	0.01	1.21	0.22
	Gender (Male vs. Female)	0.94	2.57	0.3	3.12	< 0.01
	EPPK1 (High expression vs. Low expression)	0.41	1.51	0.34	1.21	0.22
	Stage (III + IV vs. I + II)	1.61	5.01	0.42	3.8	< 0.01
	EPPK1 x Stage	-2.43	0.09	0.7	-3.48	< 0.01
LUSC	Pack Years	-0.01	0.99	0	-2.1	0.04
	Gender (Male vs. Female)	-0.03	0.97	0.23	-0.13	0.89
	EPPK1 (High expression vs. Low expression)	0.14	1.15	0.37	0.39	0.7
	Stage (III + IV vs. I + II)	0.87	2.39	0.33	2.66	0.01
	EPPK1 x Stage	0.02	1.02	0.43	0.04	0.96

EPPK1 x Stage: Interaction effect of EPPK1 and stage

Coef: Coefficient in the model

Reference level for each variable in the model: Female, Low EPPK1 expression, I + II stage

### EPPK1 protein expression in normal epithelial cells increases after exposure to cigarette smoking for 16 weeks

**Fig. 2:**
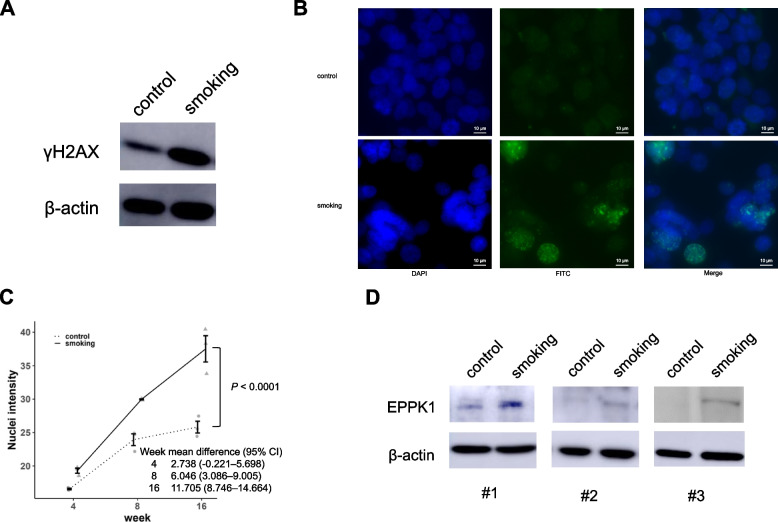
Exposure of normal bronchial epithelial cells to cigarette smoking. **A** High expression of γH2AX protein observed after 16 weeks of smoking exposure in BEAS2B. **B** Representative immunofluorescence images of γH2AX following smoking exposure for 16 weeks in BEAS2B (40x). **C** Quantification of γH2AX using immunofluorescent assays (nuclei intensity/nuclei) following 16 weeks of smoking exposure in BEAS2B. γH2AX levels increased after smoking exposure for 16 weeks (mean difference with CI = 11.705 [8.746–14.664], *P* < 0.0001). Dot plot with mean and standard error per group by week. CI: confidence interval. **D** EPPK1 protein is highly expressed after 16 weeks of smoking exposure in BEAS2B

Our clinical data revealed that patients with high expression of EPPK1 had a higher smoking pack-year history than those with low expression (Difference with CI = 10 [0.00–21.00], *P* = 0.032 in LUAD, Table 3; Difference with CI = 20 [10.00–28.00], *P* < 0.001 in LUSC, Table 4). To further investigate the impact of smoking on EPPK1 expression, we exposed BEAS2B, a normal bronchial epithelial cell line, to cigarette smoke for 16 weeks. The average smoking density and maximum carbon monoxide level during exposure were 1.8 mg/l and 121 ppm, respectively. As a positive control for smoking exposure, we initially examined γH2AX, a biomarker for DNA double-stranded breaks. We confirmed an increase in γH2AX levels after 16 weeks of smoking exposure using WB and immunofluorescence, validating the DNA damage caused by our smoking method (Fig. 2A, B, mean difference with CI = 11.705 [8.746–14.664], Fig. 2C). Subsequently, we evaluated EPPK1 expression in normal bronchial epithelial cells following 16 weeks of smoking. Our results demonstrated a correlation between EPPK1 expression and smoking (Fig. 2D), consistent with our clinical study where EPPK1 expression significantly correlated with pack-years (Tables 3 and 4).

### EPPK1 regulates epithelial to mesenchymal transition, MYC, and p53

**Fig. 3:**
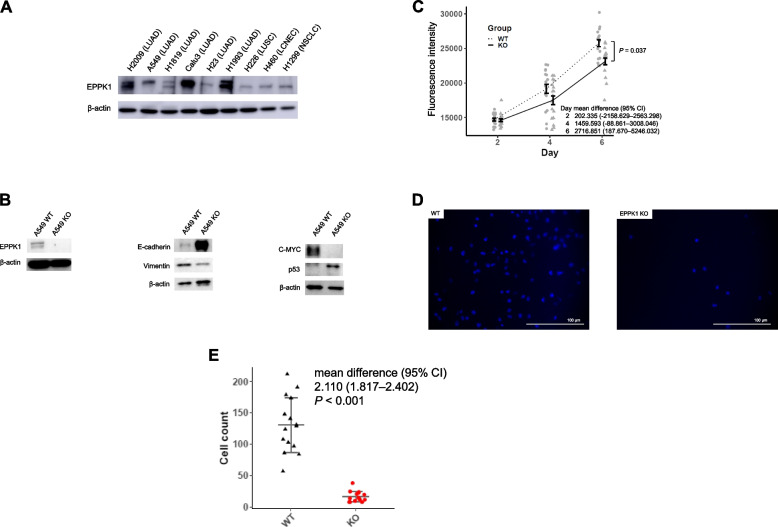
Targeting EPPK1 with CRISPR-Cas9 in A549 cells. **A** EPPK1 protein expression in lung adenocarcinoma (LUAD), lung squamous cell carcinoma (LUSC), large cell neuroendocrine carcinoma (LCNEC), and non-small cell lung cancer (NSCLC). **B** Western blotting results show that EPPK1 was knocked out in A549. The results confirmed MET due to the upregulation of E-cadherin and downregulation of Vimentin after silencing EPPK1. Further, we confirmed that oncogenes were upregulated, and tumor suppressor genes were downregulated after silencing EPPK1. **C** Cell proliferation assays reveal that cell growth inhibition was induced after silencing EPPK1 in A549 (mean difference with CI = 2716.851 [187.670–5246.032], *P* = 0.037). CI: confidence interval. **D** Representative image of invasion assays for wild type and EPPK1 KO cells in A549 (20x). **E** Inhibition of cell invasion after silencing EPPK1 in A549 (mean difference with CI = 2.110 [1.817–2.402], *P* < 0.001). Dot plot with mean and standard error per group by day. CI: confidence interval

Based on our findings indicating the critical role of EPPK1 in cancer development and its association with poor prognosis in LUAD and other cancers, we aimed to investigate whether the loss of EPPK1 function could impede cancer progression. Initially, we assessed EPPK1 protein expression in NSCLC cell lines using WB analysis (Fig. 3A). Additionally, we verified EPPK1 protein expression in our cell line microarray through IHC (Fig. S1B). To explore the impact of EPPK1 on LUAD progression, we conducted a loss-of-function study using CRISPR-Cas9 for all LUAD cell lines in Fig. 3A. We successfully generated KO of EPPK1 to investigate epithelial to mesenchymal transition (EMT), its reverse process mesenchymal to epithelial transition (MET) [21], and angiogenesis in the A549 LUAD cell line. Our analysis revealed that the KO of EPPK1 led to increased expression of E-cadherin and p53, while Vimentin, and MYC expression decreased, as demonstrated by WB analysis (Fig. 3B). These findings indicated a MET process, as evidenced by the upregulation of E-cadherin, downregulation of Vimentin, upregulation of tumor suppressor proteins, and decreased expression of oncogenic proteins following EPPK1 silencing. Overall, our results strongly suggested that EPPK1 promotes EMT and lung cancer development.

### EPPK1 regulates lung cancer cell proliferation and invasion

Based on our data indicating the association of EPPK1 with cancer development and EMT we conducted an analysis of cell growth and invasion following the KO of EPPK1. To elucidate the mechanisms through which EPPK1 regulates cell proliferation, we compared the proliferation of WT and KO cells at 2, 4, and 6 days. Our findings revealed that EPPK1 KO cells had a slower proliferation rate than WT cells (mean difference with CI = 2716.851 [187.670–5246.032], *P* = 0.037, Fig. 3C). To examine the impact of EPPK1 regulation on cell invasion, we conducted a comparison of cell invasion between WT and KO cells. The results of invasion assays demonstrated that EPPK1 KO cells exhibited a reduced invasion ability compared to that of A549 WT cells (mean difference with CI = 2.110 [1.817–2.402], *P* < 0.001, Fig. 3D, E).

### EPPK1 KO affects genomic alterations

**Fig. 4:**
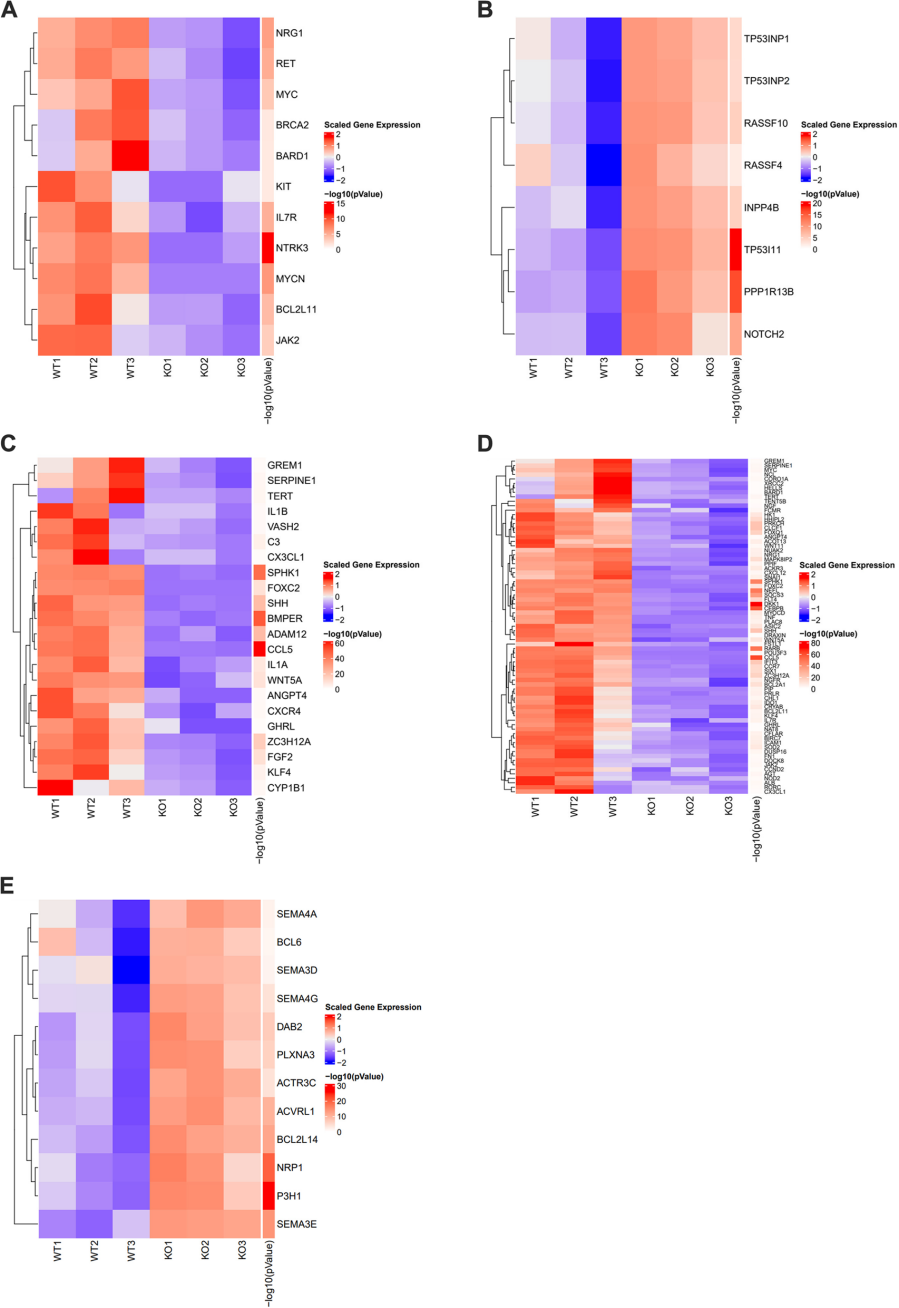
Heatmap comparing RNA expression between WT and KO of EPPK1 in A549 cells. **A** Heatmap demonstrates that oncogenes were downregulated after silencing EPPK1 in A549 cells. The color scale relates to RNA expression levels: red, high; white, normal; blue, low expression. **B** RNA seq results demonstrate that the expression of tumor suppressor genes is upregulated after silencing EPPK1 in A549 cells. **C** RNA seq results demonstrate that angiogenesis genes were downregulated after silencing EPPK1 in A549 cells. **D** RNA seq results demonstrate that the expression of anti-apoptosis genes was downregulated after silencing EPPK1 in A549 cells. E RNA seq results demonstrate that the expression of anti-cell growth genes was upregulated after silencing EPPK1 in A549 cells

**Fig. 5:**
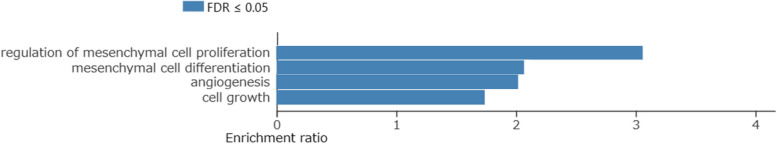
GO enrichment analysis of selected molecular functions comparing RNA profiles between WT and KO of EPPK1 in A549 cells. GO enrichment analysis revealed that WT cells were enriched in functions related to the regulation of mesenchymal cell proliferation, mesenchymal differentiation, angiogenesis, and cell growth compared to those in KO cells. GO gene ontology, FDR false discovery rate

Based on our accumulated evidence indicating the involvement of EPPK1 in cancer development, EMT, cell proliferation, and cell invasion, we employed RNA sequencing to examine the expression levels of WT and KO EPPK1. Out of the 15,282 genes analyzed, 2,201 (14%) exhibited alterations following EPPK1 KO. Specifically, after KO, the expression of 11 oncogenes, 75 anti-apoptosis genes, and 22 angiogenesis genes was downregulated, while the expression of 8 tumor suppressor genes and 12 anti-cell growth genes was upregulated (Fig. 4A–D). Furthermore, performing GO enrichment analysis on selected molecular functions of differentially expressed genes between WT and KO cells, we observed that WT cells exhibited enhanced regulation of mesenchymal cell proliferation, mesenchymal differentiation, angiogenesis, and cell growth in comparison to those in KO cells (Fig. 5). These results serve to underscore the involvement of EPPK1 in tumorigenesis, EMT, angiogenesis, and cell growth.

## Discussion

In this study, we investigated the association between EPPK1 and smoking exposure in surgically resected lung tissue. We found that EPPK1 expression is correlated with smoking pack years, and we were able to replicate these effects by exposing normal bronchial epithelial cell lines to smoking (Figs. 1B and 2A–D, Tables 3 and 4). Furthermore, our findings indicated that patients with higher levels of EPPK1 expression, particularly those with early-stage LUAD, had worse OS. Additionally, we observed that EPPK1 KO resulted in decreased invasion and proliferation, changes in proteins associated with EMT signaling, and differential expression of key oncogenic, angiogenic, apoptotic, tumor suppressor, and anti-cell growth genes (Figs. 3B, 4 and 5).

The main focus of this study was to examine the role of EPPK1 in the development of LUAD, specifically in early-stage LUAD. We investigated the functional and clinical significance of EPPK1 in normal bronchial cells and LUAD. Smoking is a well-known risk factor for lung cancer [22] due to its ability to cause DNA damage, as indicated by the presence of γH2AX [23] and genomic instability, which can contribute to tumorigenesis [24]. Our previous research demonstrated that smoking could influence genomic features involved in tumorigenesis [25]. Our results showed that smoking upregulates EPPK1 expression in normal bronchial epithelial cells and that high EPPK1 expression is associated with smoking exposure (pack-years) and early-stage LUAD. We conclude that smoking-induced DNA damage and genomic instability contribute to increased EPPK1 expression in normal bronchial epithelial cells, potentially promoting lung cancer development.

We also investigated the expression of EPPK1 in multiple cancer types compared to that in normal tissues (Fig. 1A) and its role in the survival of various cancers and early-stage LUAD (Table 1, Fig. 1C, Table 5). Previous reports have described the association of EPPK1 with poor prognosis in hepatocellular cancer and esophageal squamous cell cancer [26, 27], as well as its potential as a biomarker for pancreatic and cervical cancer [28, 29]. However, the specific role of EPPK1 in lung cancer has not been explored. Our results, comparing EPPK1 mRNA expression between cancer and normal tissues, support the evidence suggesting that EPPK1 is associated with poor prognosis in liver and esophageal cancers [26, 27]. Moreover, our findings indicate that elevated EPPK1 protein expression is associated with poor survival in stage I and stages I and II LUAD, highlighting the crucial role of EPPK1 in progression and suggesting its potential as a prognostic biomarker in LUAD.

To gain a better understanding of the role of EPPK1 in LUAD, we conducted functional and genomic analyses using CRISPR-Cas9 to KO EPPK1 in LUAD cell lines. Our results confirmed the relationship between EPPK1 and EMT signaling (Fig. 3B), as well as its involvement in the regulation of onco/suppressor genes, angiogenesis, cell invasion, and cell growth (Fig. 3C–E). This suggests that EPPK1 plays a crucial role in the progression of lung cancer through the EMT signaling pathway, which is associated with tumorigenesis [30]. Furthermore, RNA sequencing analysis, including GO enrichment analysis of selected molecular functions, indicated that EPPK1 mRNA is associated with lung cancer development (Figs. 4A–E and 5). We also observed downregulation of MYC and upregulation of p53 expression at both the protein and RNA levels after EPPK1 KO. MYC and p53 regulate each other and can induce cell cycle arrest in the G1 or G2 phase, representing an early event in tumorigenesis resulting from genomic instability [31–33]. Therefore, our results suggest that EPPK1 plays a crucial role in the regulation of cancer development in LUAD through its effects on multiple signaling pathways and genes.

While our study provided multiple insights into EPPK1, certain limitations need to be acknowledged. First, the clinical part of our study was retrospective, emphasizing the need for prospective randomized controlled trials to validate these results. Second, although we integrated data for patients with NSCLC regarding mRNA and immunohistochemical expression from two databases in the clinical part, conducting a study using an identical database would be valuable for result comparison. Third, although cigarette smoking exposure correlated with EPPK1 expression in a normal bronchial epithelial cell, it didn’t mean that smoking-induced EPPK1 directly led to LUAD. Fourth, despite combining clinical and in vitro studies in this research, additional in vivo studies are necessary to fully elucidate the role and significance of EPPK1.

## Conclusion

Our study provides evidence that EPPK1 plays a vital role in the development and progression of LUAD. We demonstrated that smoking-induced inflammation, DNA damage, and genomic instability contribute to increased EPPK1 expression in normal bronchial epithelial cells, potentially promoting lung cancer development. Furthermore, we found that high EPPK1 expression is associated with worse OS, particularly in early-stage LUAD. Our results suggest that EPPK1 holds promise as a prognostic biomarker for LUAD. Lastly, we demonstrated that EPPK1 plays a crucial role in the regulation of EMT and cancer development in LUAD by affecting multiple signaling pathways and genes. Overall, our study emphasizes the importance of EPPK1 as a potential target for the development of new therapeutic strategies for LUAD. Further studies are needed to elucidate the crosstalk between EPPK1 and cancer-related MYC and p53 pathways during development and tumorigenesis.

### Supplementary Information

**Supplementary Material 1.**

**Supplementary Material 2.**

**Supplementary Material 3.**

**Supplementary Material 4.**

## Data Availability

No datasets were generated or analysed during the current study.

## Abbreviations

ATM: Ataxia telangiectasia mutated
CI: Confidence interval
DMEM: Dulbecco’s modified Eagle’s medium
EP300: E1A binding protein P300
EPPK1: Epiplakin 1
HR: Hazards ratio
IHC: Immunohistochemistry
KMT2C: Lysine methyltransferase 2C
KO: Knockout
LUAD: Lung adenocarcinoma
LUSC: Lung squamous cell carcinoma
EMT: Epithelial-to-mesenchymal transition
MET: Mesenchymal to epithelial transition
NSCLC: Non-small cell lung cancer
OS: Overall survival
PIK3CG: Phosphatidylinositol-4,5-bisphosphate 3-kinase catalytic subunit gamma
TCGA: The Cancer Genome Atlas
TMA: Tissues microarray
WB: Western blotting
