# Supplementary material for: Elucidating the role of EPPK1 in lung adenocarcinoma development
Source: BMC Cancer. 2024 Apr 10;24:441. doi: 10.1186/s12885-024-12185-x (PMC11005125; doi:10.1186/s12885-024-12185-x)
Supplement: Supplementary file 1 — Supplementary Material 1. [file 12885_2024_12185_MOESM1_ESM.pptx]

## Slide 1
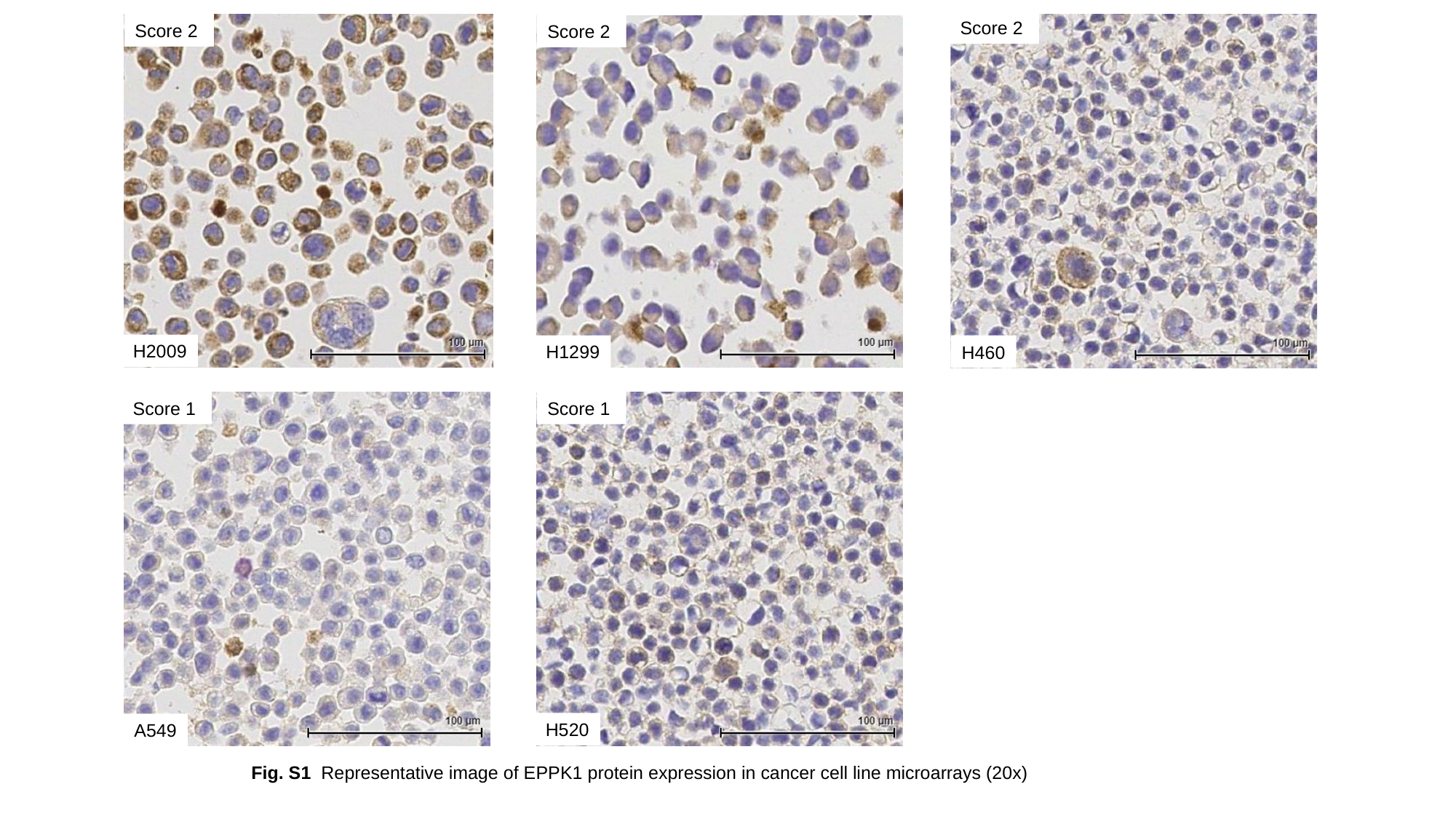

Score 2
Score 2
Score 2
H2009
H1299
H460
Score 1
Score 1
H520
A549
 Fig. S1 Representative image of EPPK1 protein expression in cancer cell line microarrays (20x)
