# Supplementary material for: Elucidating the role of EPPK1 in lung adenocarcinoma development
Source: BMC Cancer. 2024 Apr 10;24:441. doi: 10.1186/s12885-024-12185-x (PMC11005125; doi:10.1186/s12885-024-12185-x)
Supplement: Supplementary file 2 — Supplementary Material 2. [file 12885_2024_12185_MOESM2_ESM.pptx]

## Slide 1
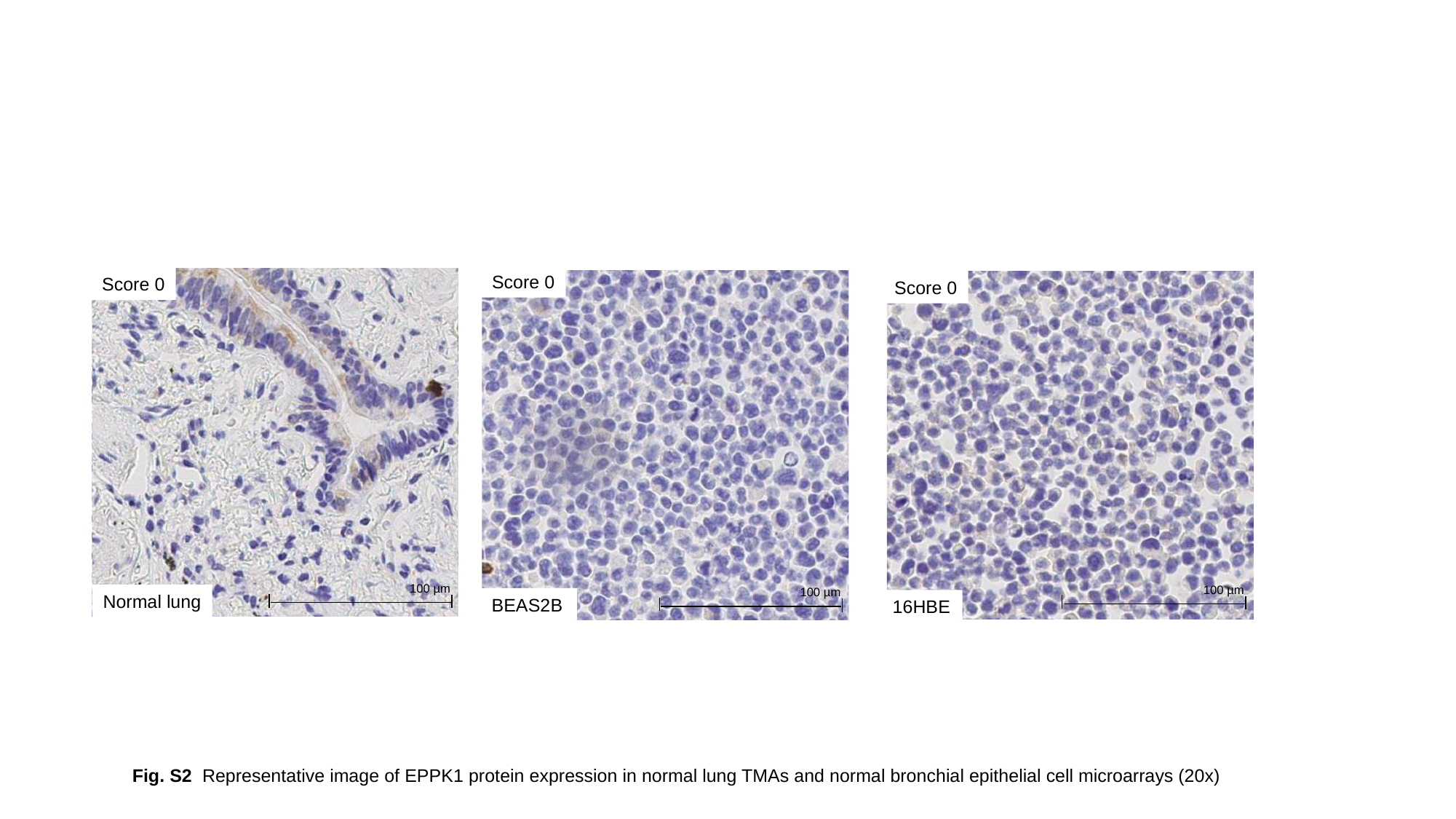

Score 0
Score 0
Score 0
100 µm
100 µm
100 µm
Normal lung
BEAS2B
16HBE
Fig. S2 Representative image of EPPK1 protein expression in normal lung TMAs and normal bronchial epithelial cell microarrays (20x)
