# Supplementary figures and images for: Elucidating the role of EPPK1 in lung adenocarcinoma development
Source: BMC Cancer. 2024 Apr 10;24:441. doi: 10.1186/s12885-024-12185-x (PMC11005125; doi:10.1186/s12885-024-12185-x)

## Slide 1
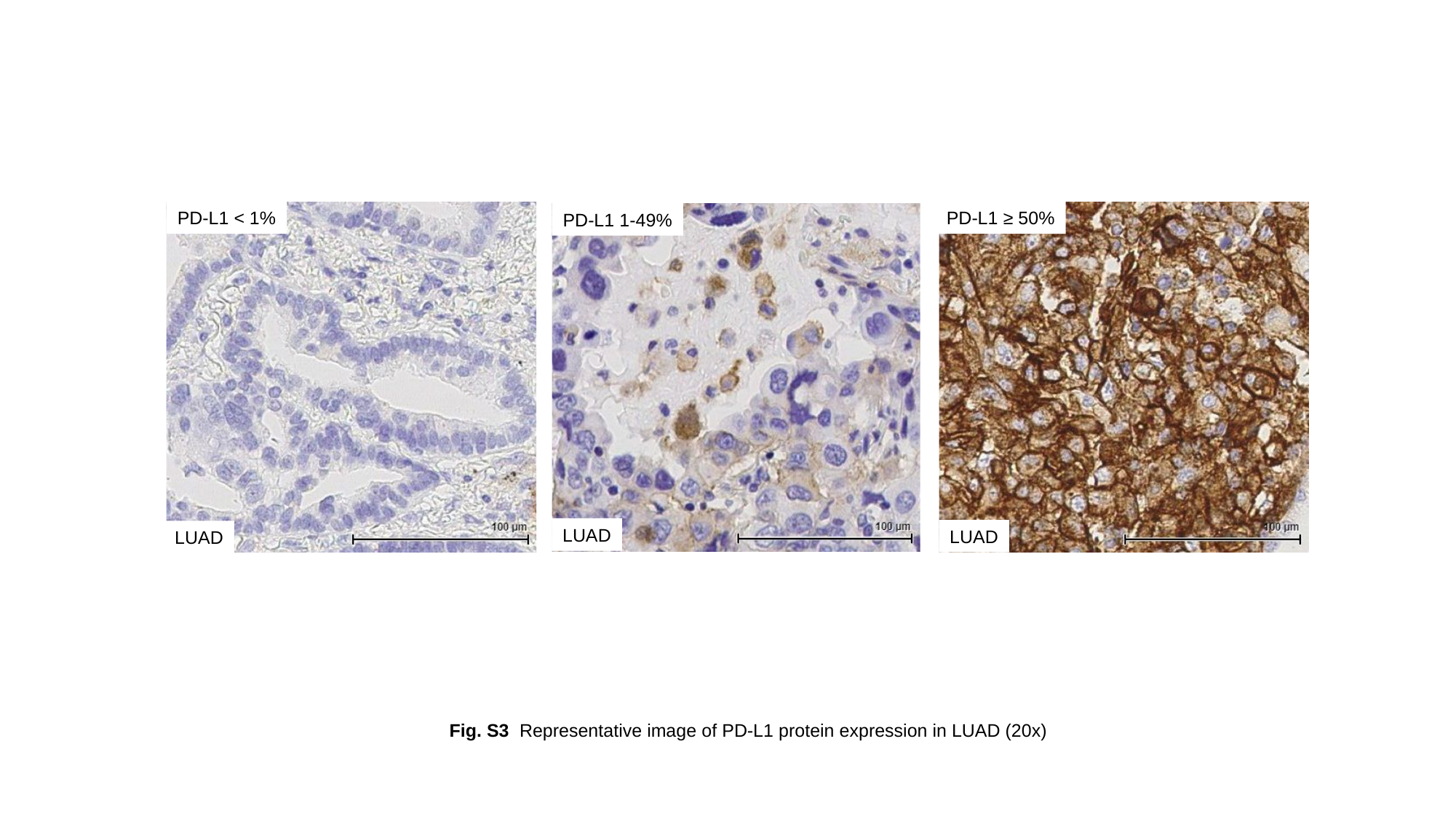

PD-L1 < 1%
PD-L1 ≥ 50%
PD-L1 1-49%
LUAD
LUAD
LUAD
Fig. S3 Representative image of PD-L1 protein expression in LUAD (20x)

Supplement: Supplementary file 3 — Supplementary Material 3. [file 12885_2024_12185_MOESM3_ESM.pptx]
