# Supplementary material for: Elucidating the role of EPPK1 in lung adenocarcinoma development
Source: BMC Cancer. 2024 Apr 10;24:441. doi: 10.1186/s12885-024-12185-x (PMC11005125; doi:10.1186/s12885-024-12185-x)
Supplement: Supplementary file 4 — Supplementary Material 4. [file 12885_2024_12185_MOESM4_ESM.pptx]

## Slide 1
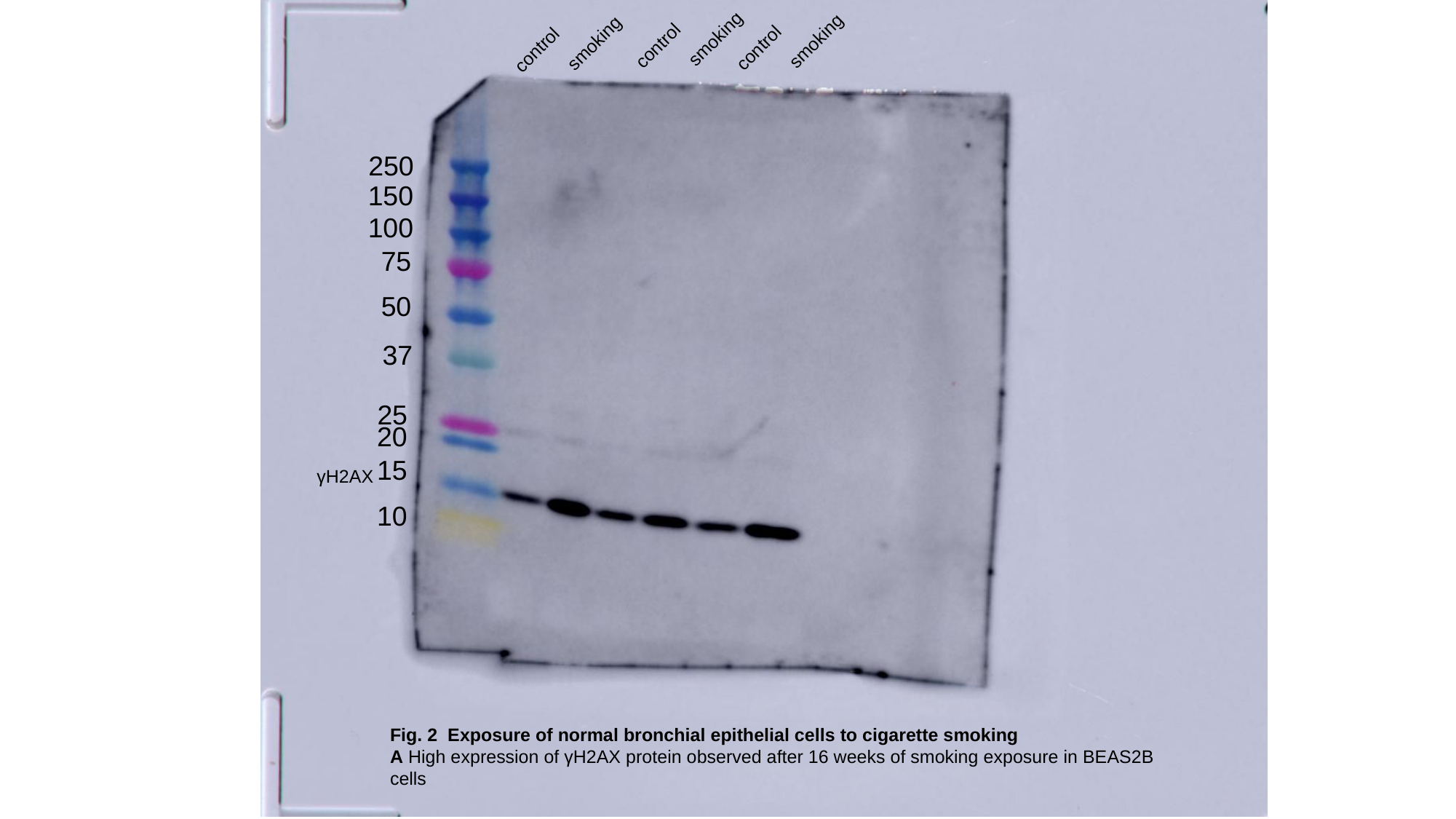

smoking
smoking
smoking
control
control
control
smoking
control
γH2AX
250
150
100
75
50
37
25
β-actin
20
15
γH2AX
10
Fig. 2 Exposure of normal bronchial epithelial cells to cigarette smoking
A High expression of γH2AX protein observed after 16 weeks of smoking exposure in BEAS2B cells

## Slide 2
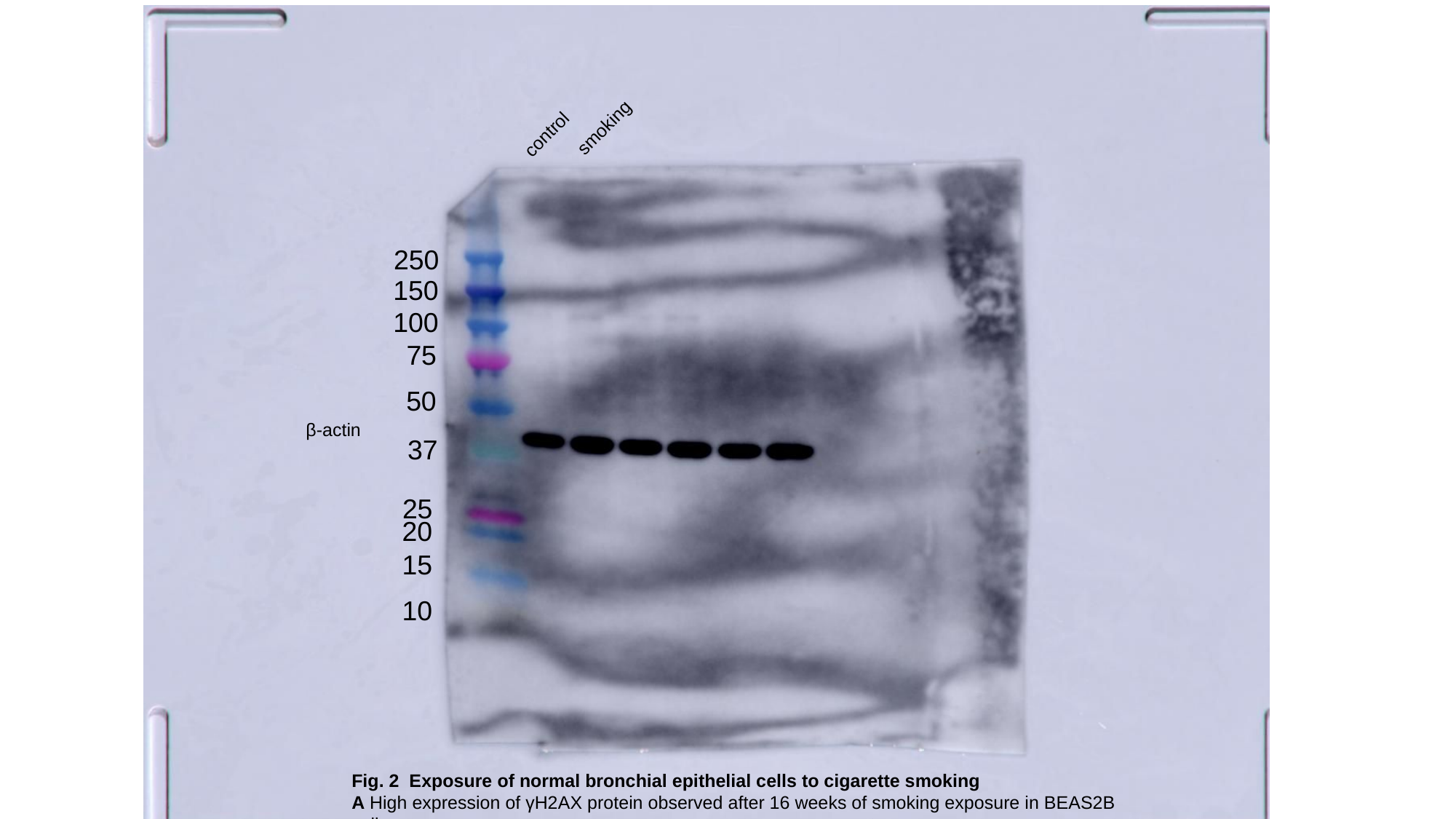

smoking
control
250
150
100
75
50
β-actin
37
25
20
15
10
Fig. 2 Exposure of normal bronchial epithelial cells to cigarette smoking
A High expression of γH2AX protein observed after 16 weeks of smoking exposure in BEAS2B cells

## Slide 3
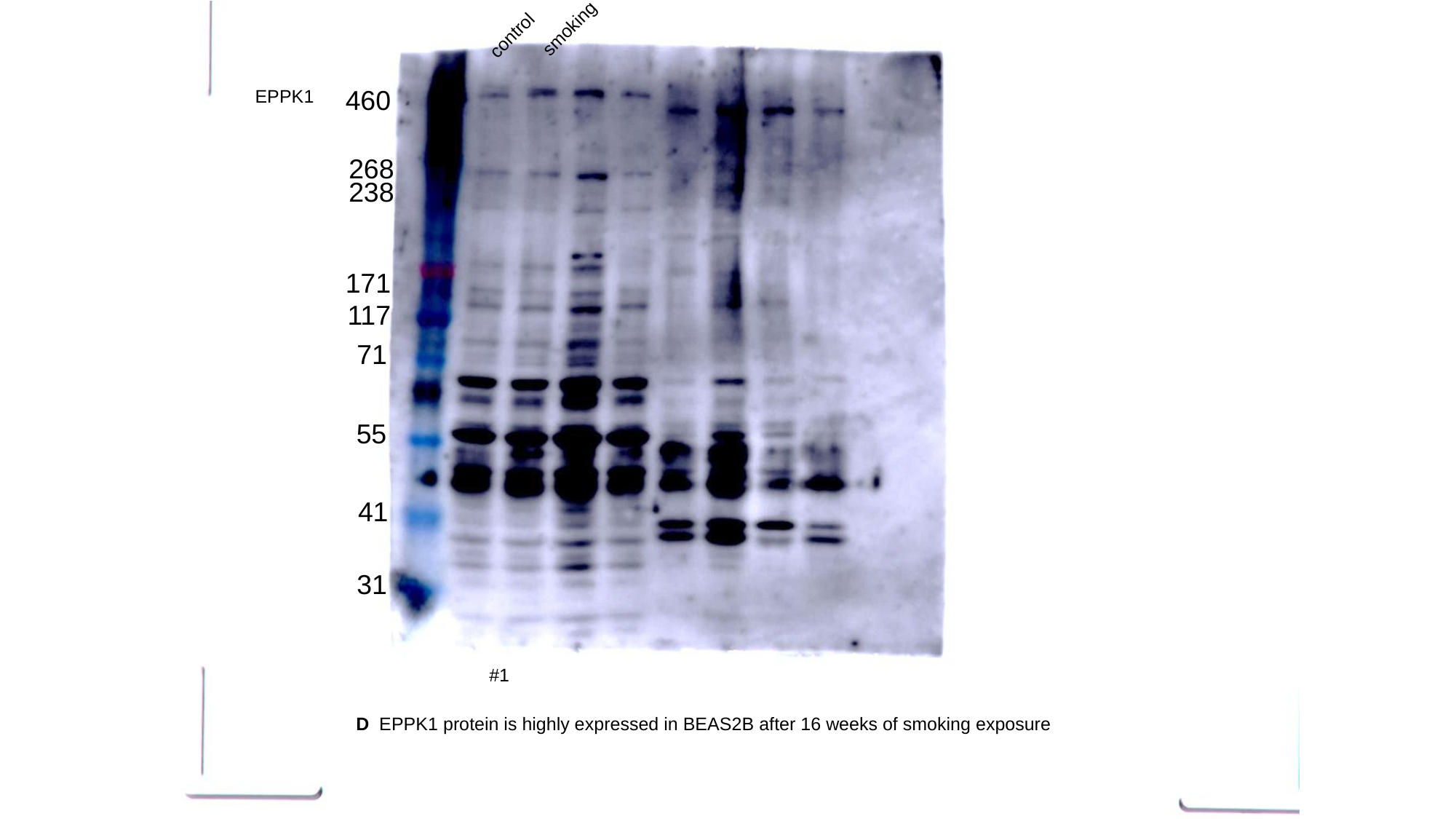

smoking
control
460
EPPK1
268
238
171
117
71
55
41
31
#1
D EPPK1 protein is highly expressed in BEAS2B after 16 weeks of smoking exposure

## Slide 4
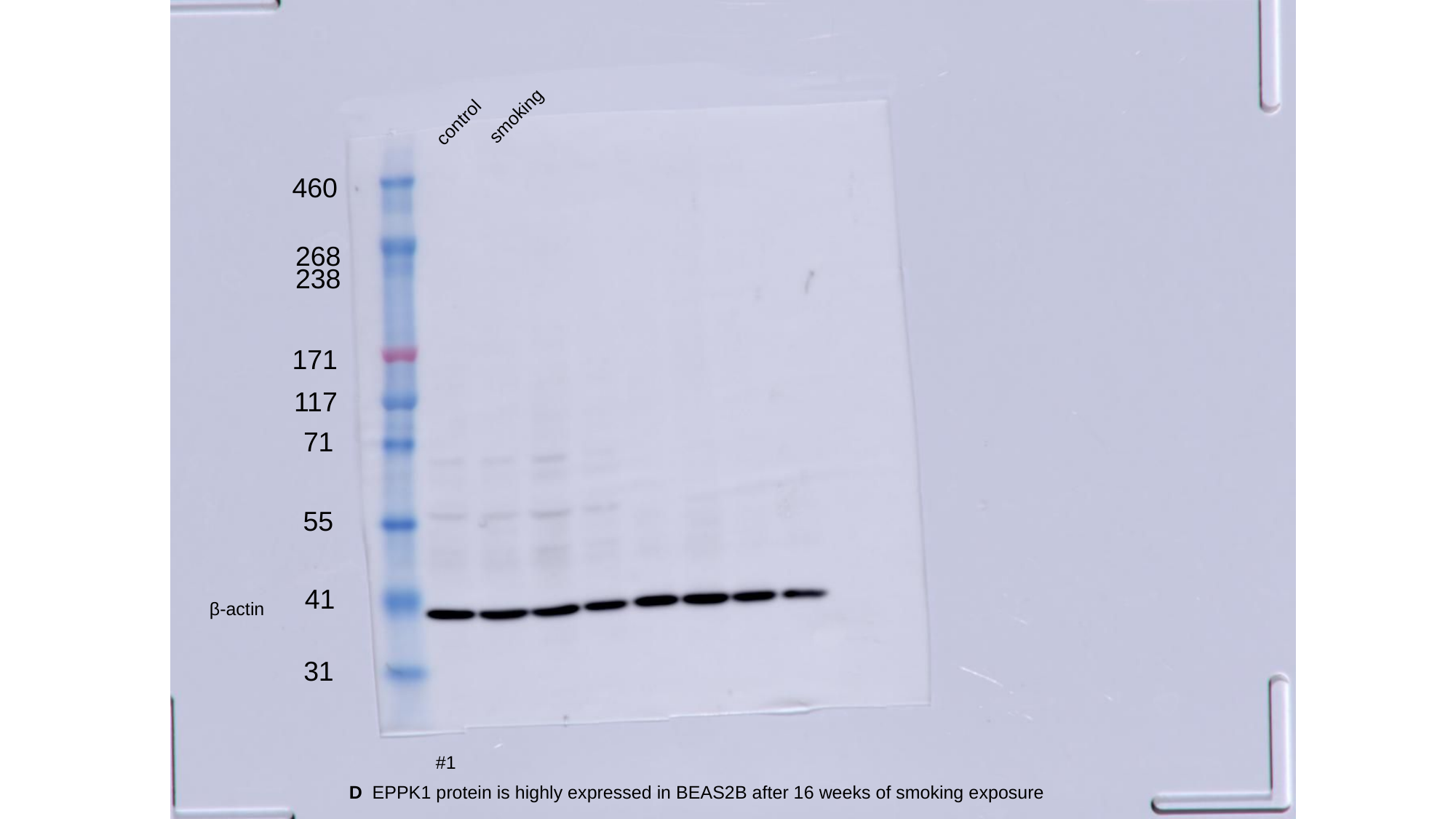

smoking
control
460
268
238
171
117
71
55
41
β-actin
31
#1
D EPPK1 protein is highly expressed in BEAS2B after 16 weeks of smoking exposure

## Slide 5
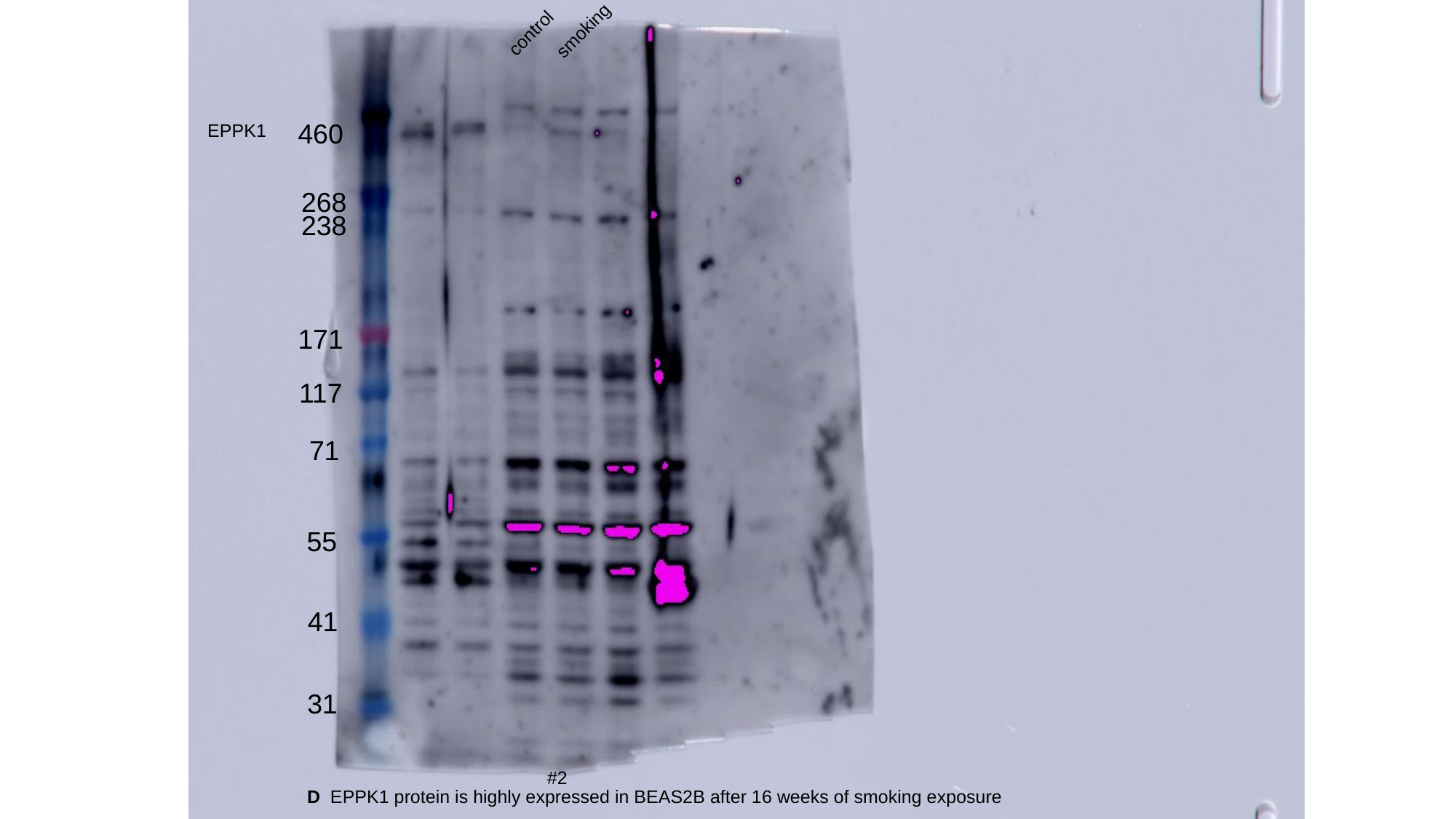

smoking
control
460
EPPK1
268
238
171
117
71
55
41
D EPPK1 protein is highly expressed in BEAS2B after 16 weeks of smoking exposure
31
#2
D EPPK1 protein is highly expressed in BEAS2B after 16 weeks of smoking exposure

## Slide 6
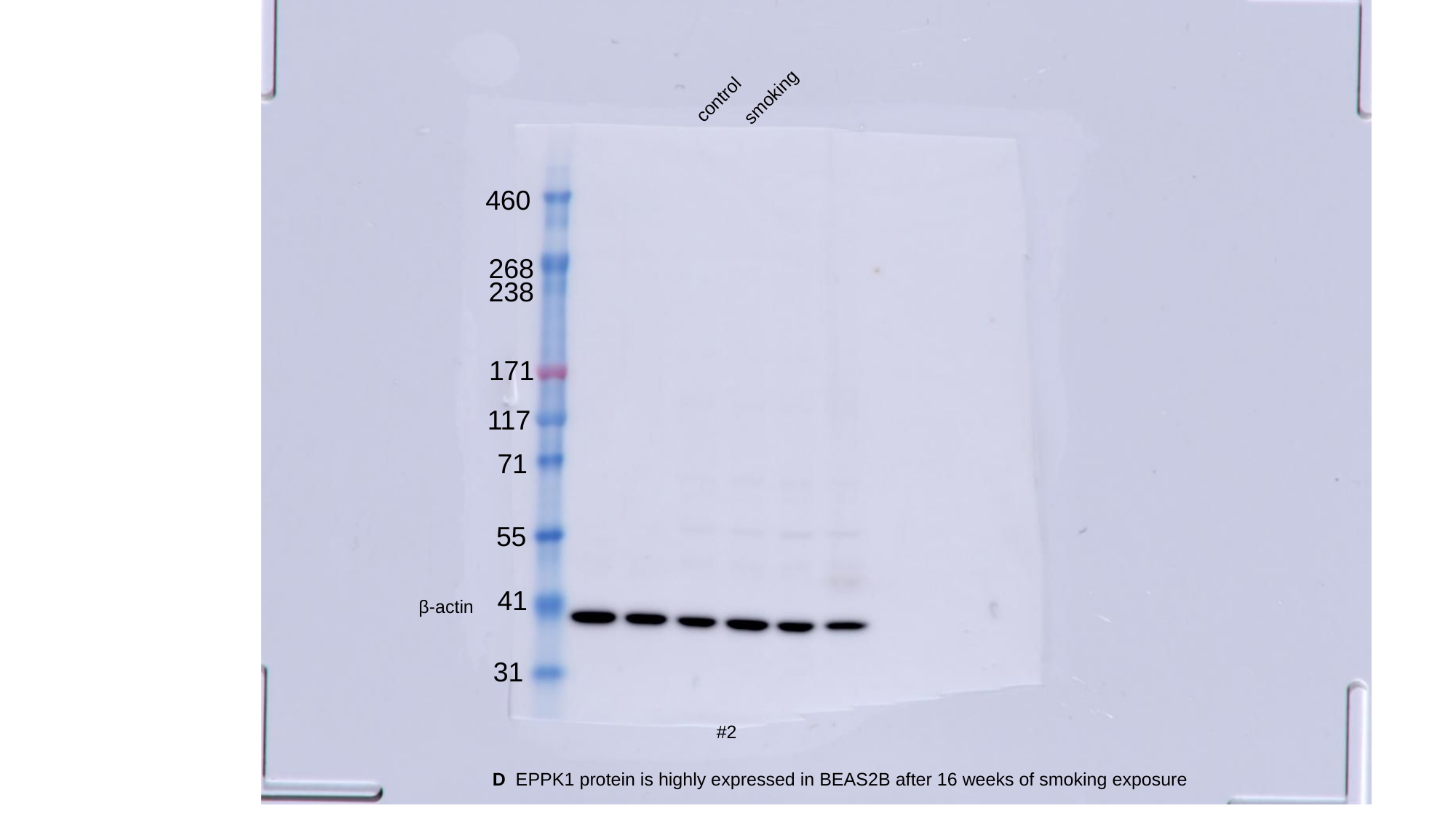

smoking
control
460
268
238
171
117
71
55
41
β-actin
31
D EPPK1 protein is highly expressed in BEAS2B after 16 weeks of smoking exposure
#2
D EPPK1 protein is highly expressed in BEAS2B after 16 weeks of smoking exposure

## Slide 7
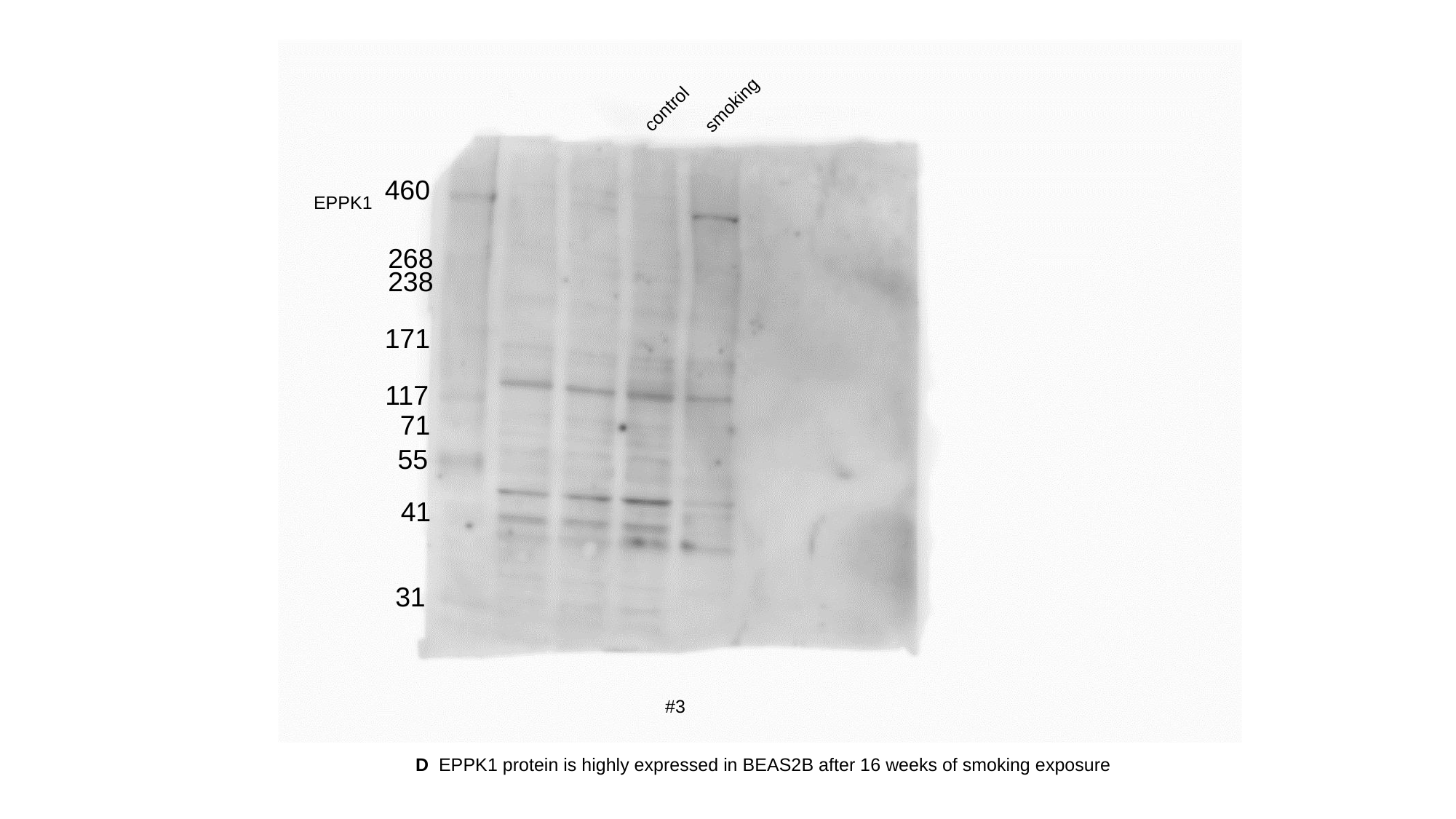

smoking
control
460
EPPK1
268
238
171
117
71
55
41
31
#3
D EPPK1 protein is highly expressed in BEAS2B after 16 weeks of smoking exposure

## Slide 8
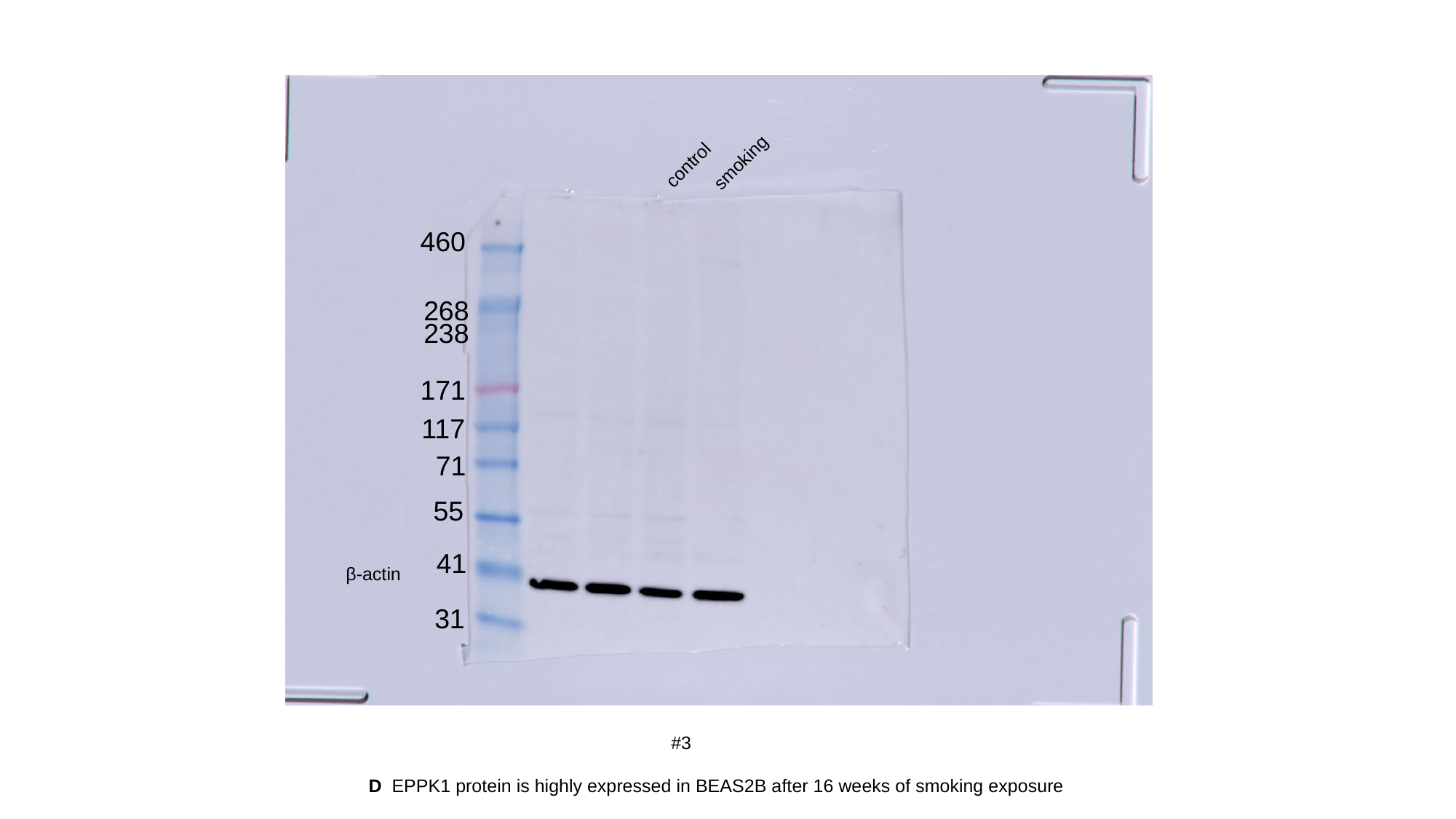

smoking
control
460
268
238
171
117
71
55
41
β-actin
31
#3
D EPPK1 protein is highly expressed in BEAS2B after 16 weeks of smoking exposure

## Slide 9
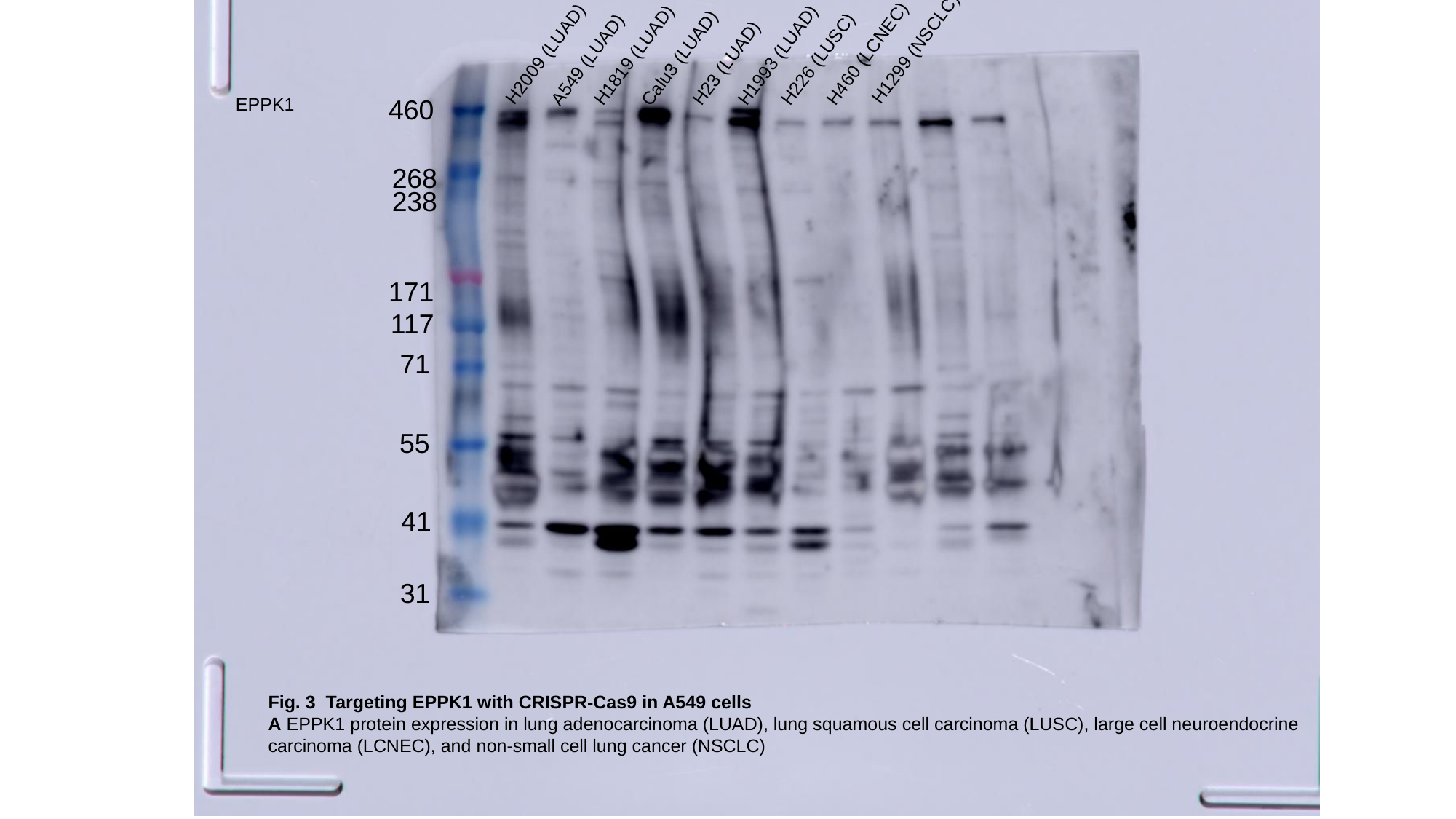

A549 (LUAD)
H1299 (NSCLC)
H460 (LCNEC)
H2009 (LUAD)
H1819 (LUAD)
H1993 (LUAD)
H226 (LUSC)
Calu3 (LUAD)
H23 (LUAD)
460
EPPK1
268
238
171
117
71
55
41
31
Fig. 3 Targeting EPPK1 with CRISPR-Cas9 in A549 cells
A EPPK1 protein expression in lung adenocarcinoma (LUAD), lung squamous cell carcinoma (LUSC), large cell neuroendocrine carcinoma (LCNEC), and non-small cell lung cancer (NSCLC)
β-actin

## Slide 10
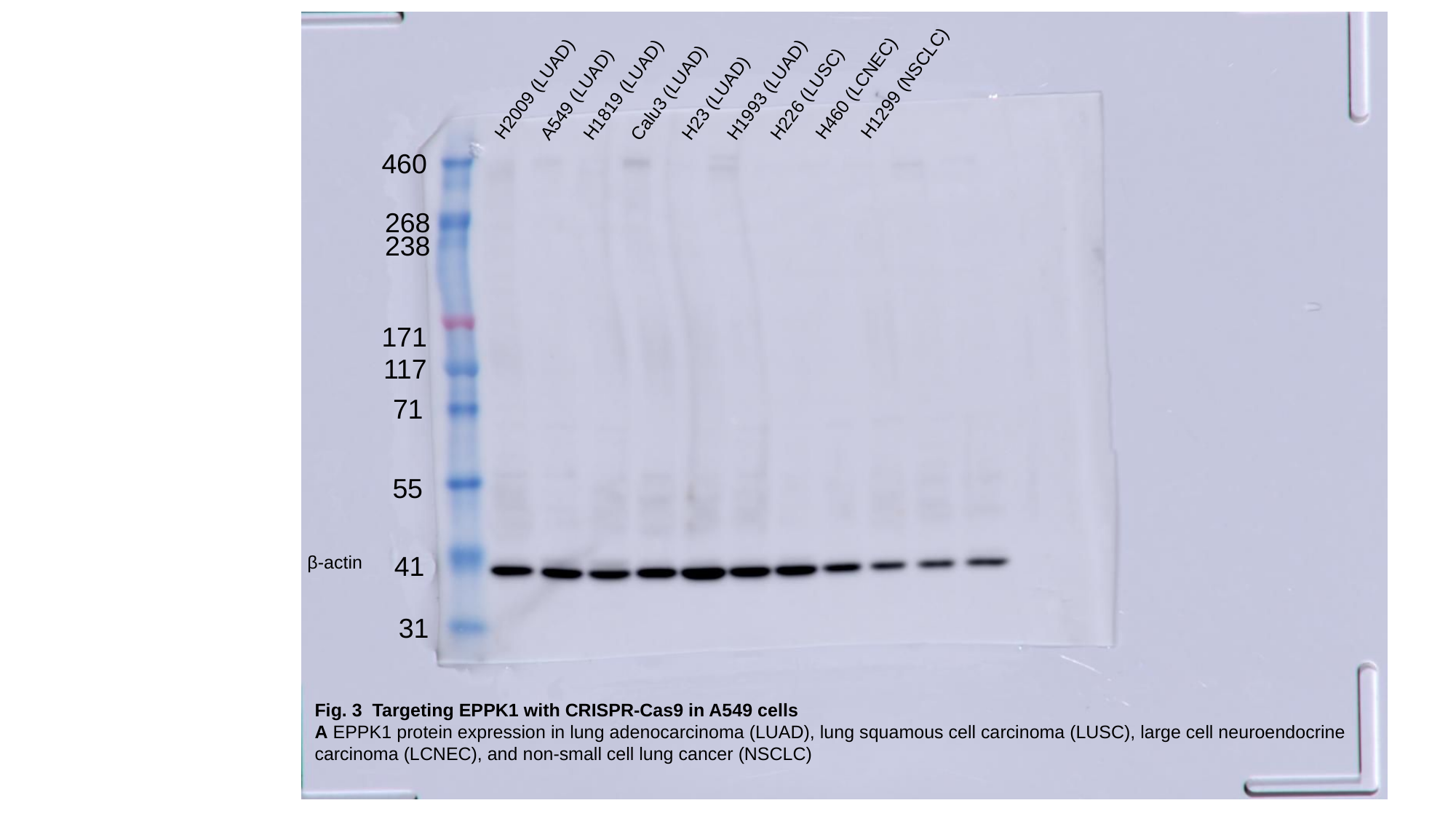

A549 (LUAD)
H1299 (NSCLC)
H460 (LCNEC)
H2009 (LUAD)
H1819 (LUAD)
H1993 (LUAD)
H226 (LUSC)
Calu3 (LUAD)
H23 (LUAD)
460
268
238
171
117
71
55
41
β-actin
31
Fig. 3 Targeting EPPK1 with CRISPR-Cas9 in A549 cells
A EPPK1 protein expression in lung adenocarcinoma (LUAD), lung squamous cell carcinoma (LUSC), large cell neuroendocrine carcinoma (LCNEC), and non-small cell lung cancer (NSCLC)

## Slide 11
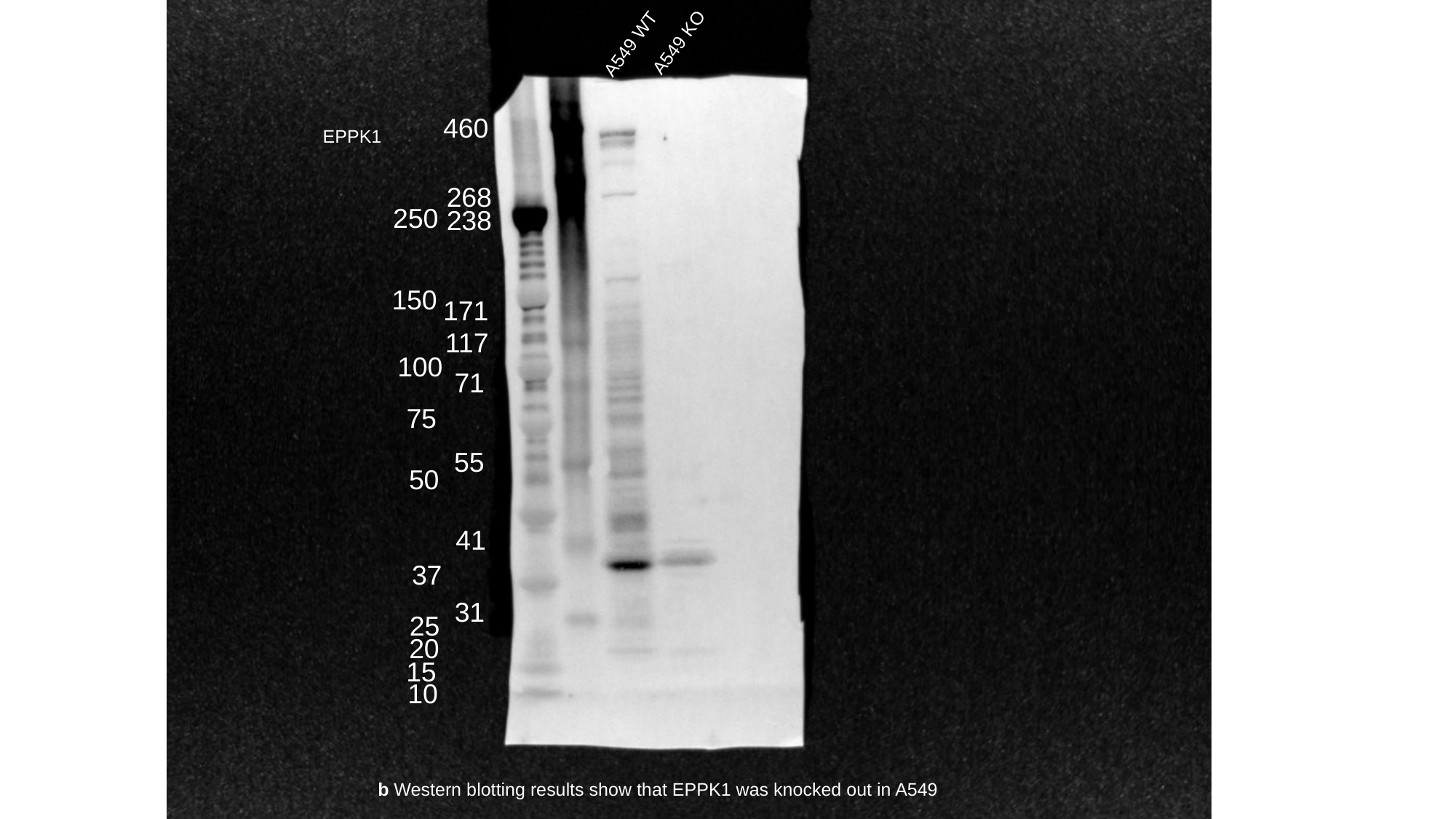

A549 KO
A549 WT
460
EPPK1
268
250
238
150
171
117
100
71
75
55
50
41
37
31
25
20
15
10
b Western blotting results show that EPPK1 was knocked out in A549

## Slide 12
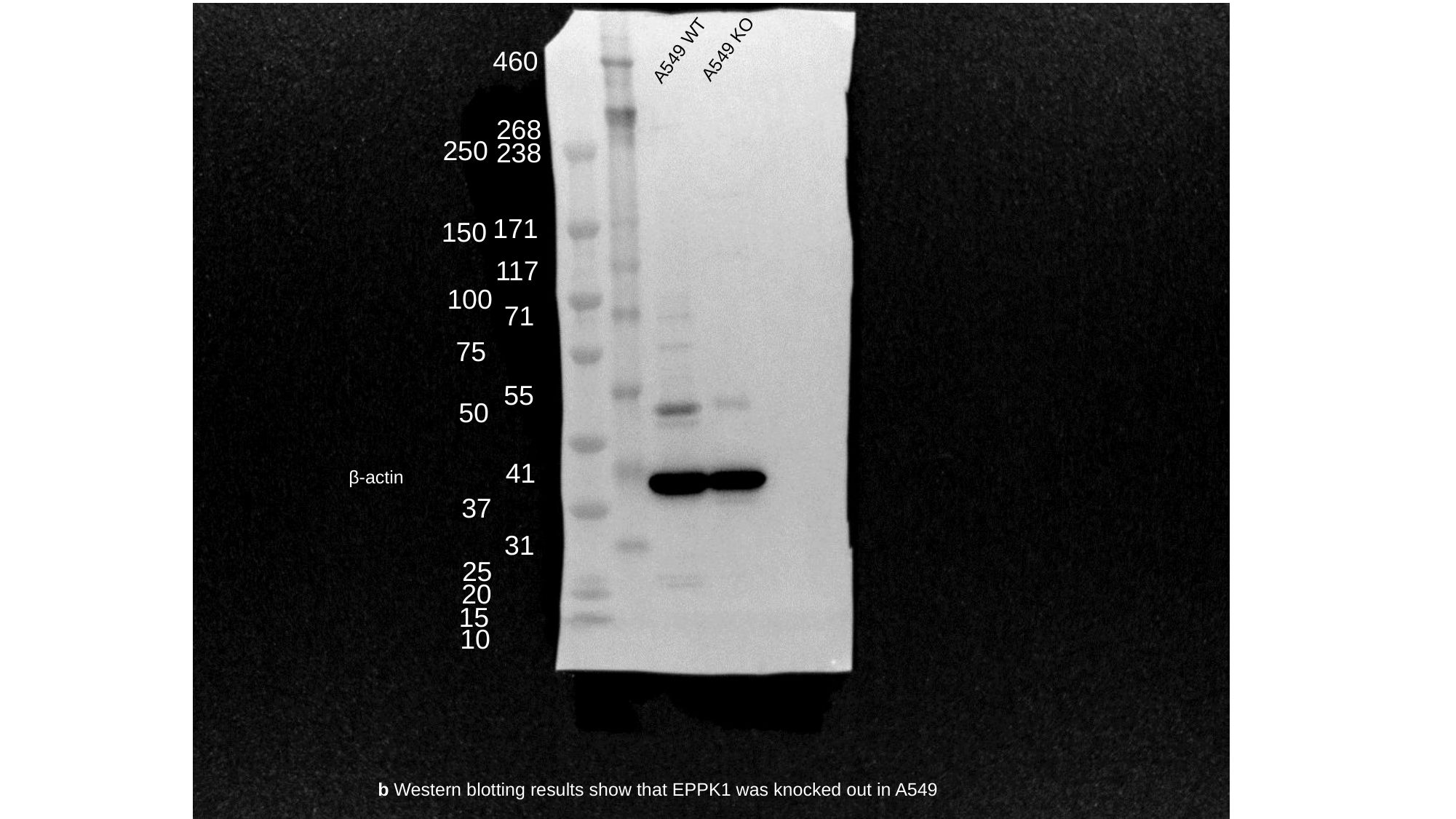

A549 KO
A549 WT
460
268
250
238
171
150
117
100
71
75
55
50
41
β-actin
37
31
25
20
15
10
b Western blotting results show that EPPK1 was knocked out in A549

## Slide 13
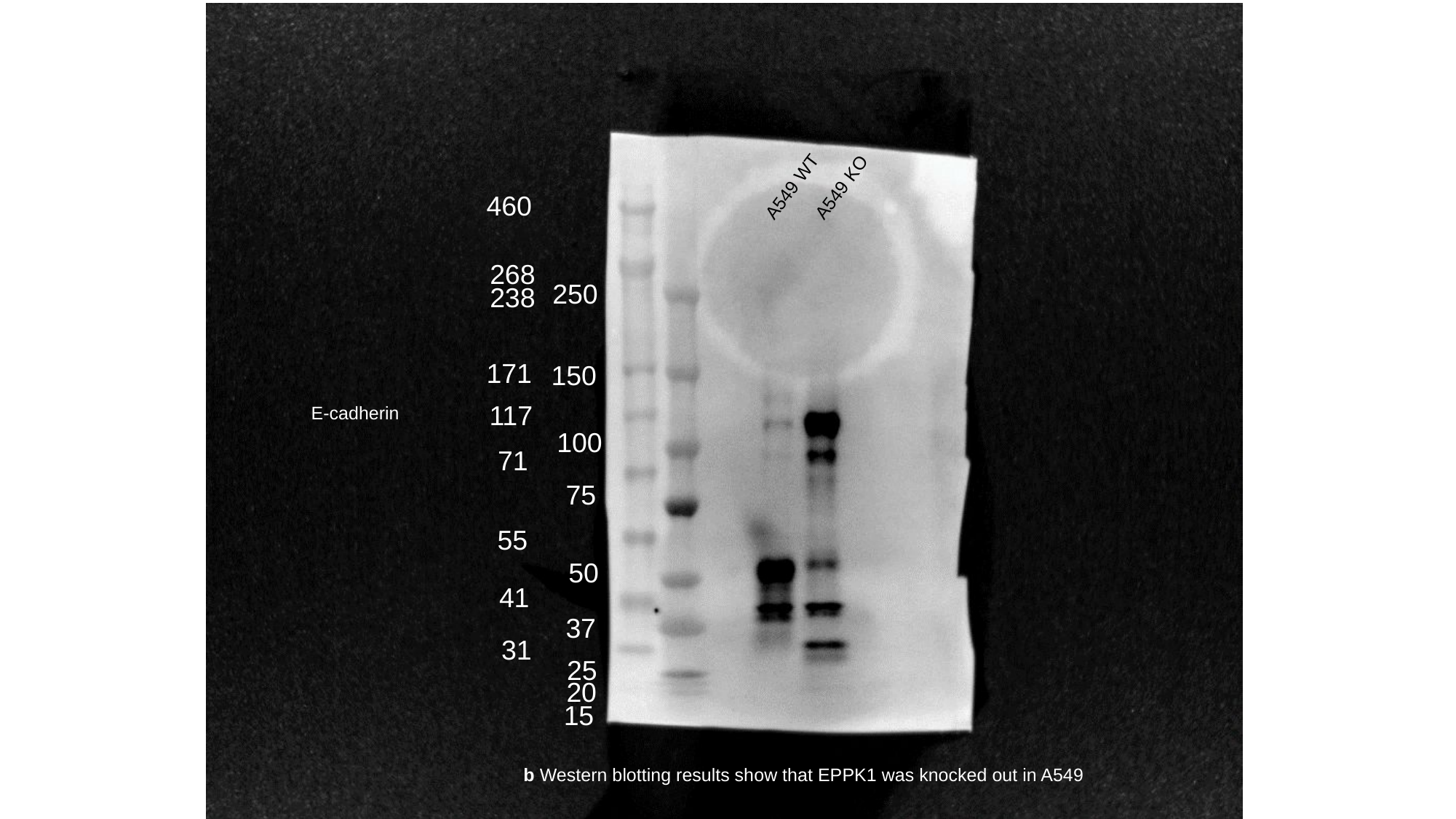

A549 WT
A549 KO
460
268
250
238
171
150
117
E-cadherin
100
71
75
55
50
41
37
31
25
20
15
b Western blotting results show that EPPK1 was knocked out in A549

## Slide 14
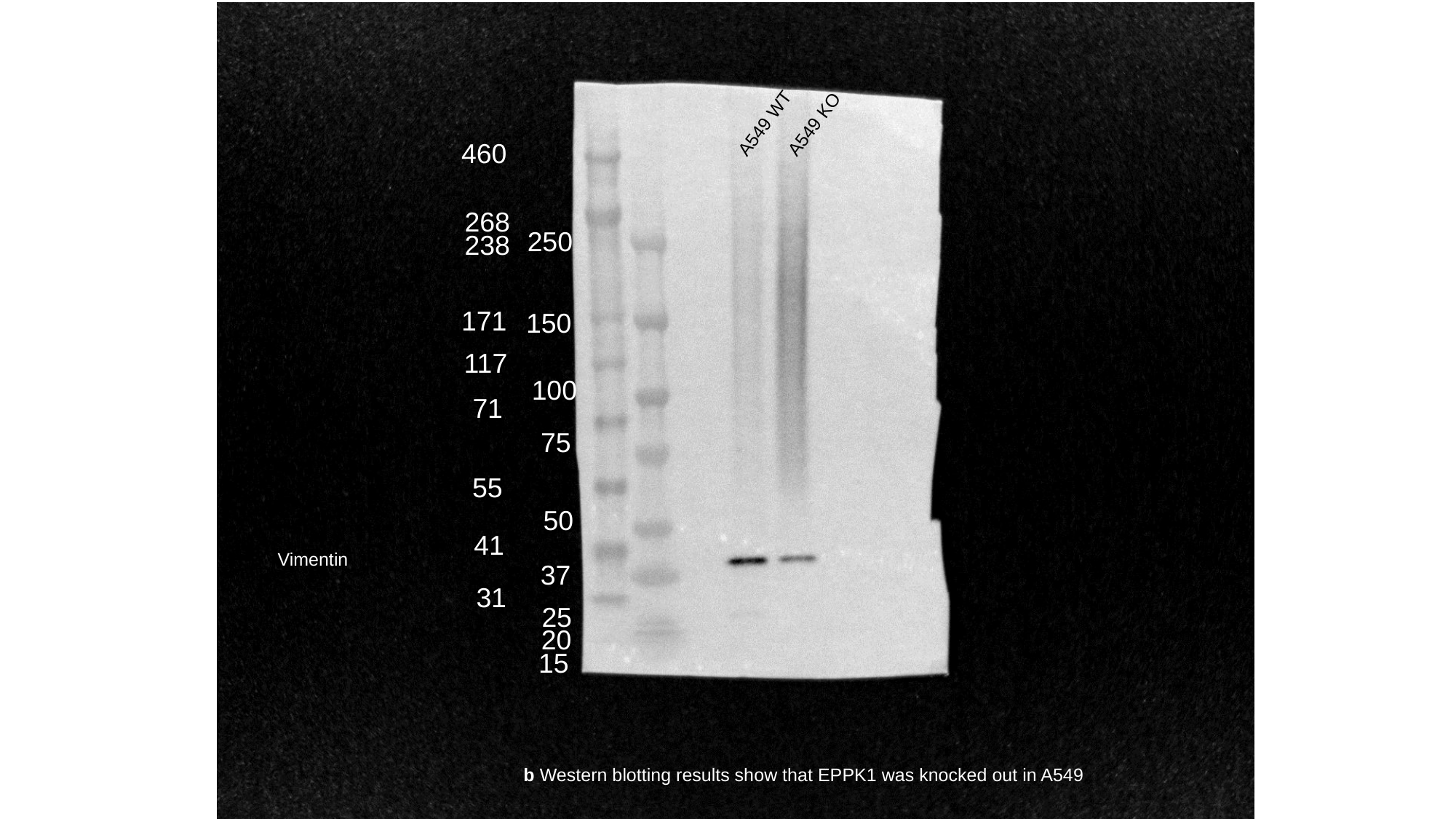

A549 WT
A549 KO
460
268
250
238
171
150
117
100
71
75
55
50
41
Vimentin
37
31
25
20
15
b Western blotting results show that EPPK1 was knocked out in A549

## Slide 15
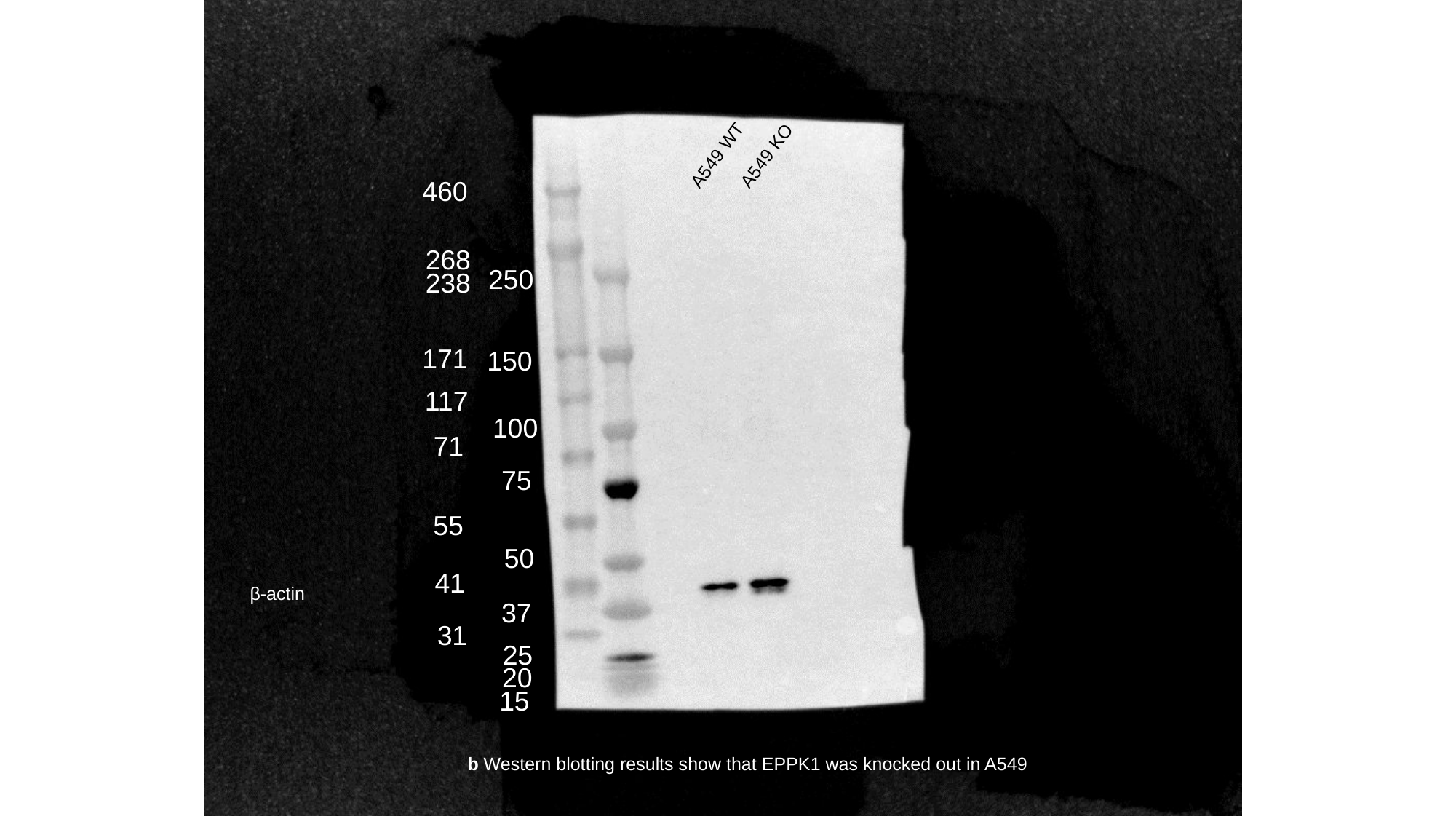

A549 WT
A549 KO
460
268
250
238
171
150
117
100
71
75
55
50
41
β-actin
37
31
25
20
15
b Western blotting results show that EPPK1 was knocked out in A549

## Slide 16
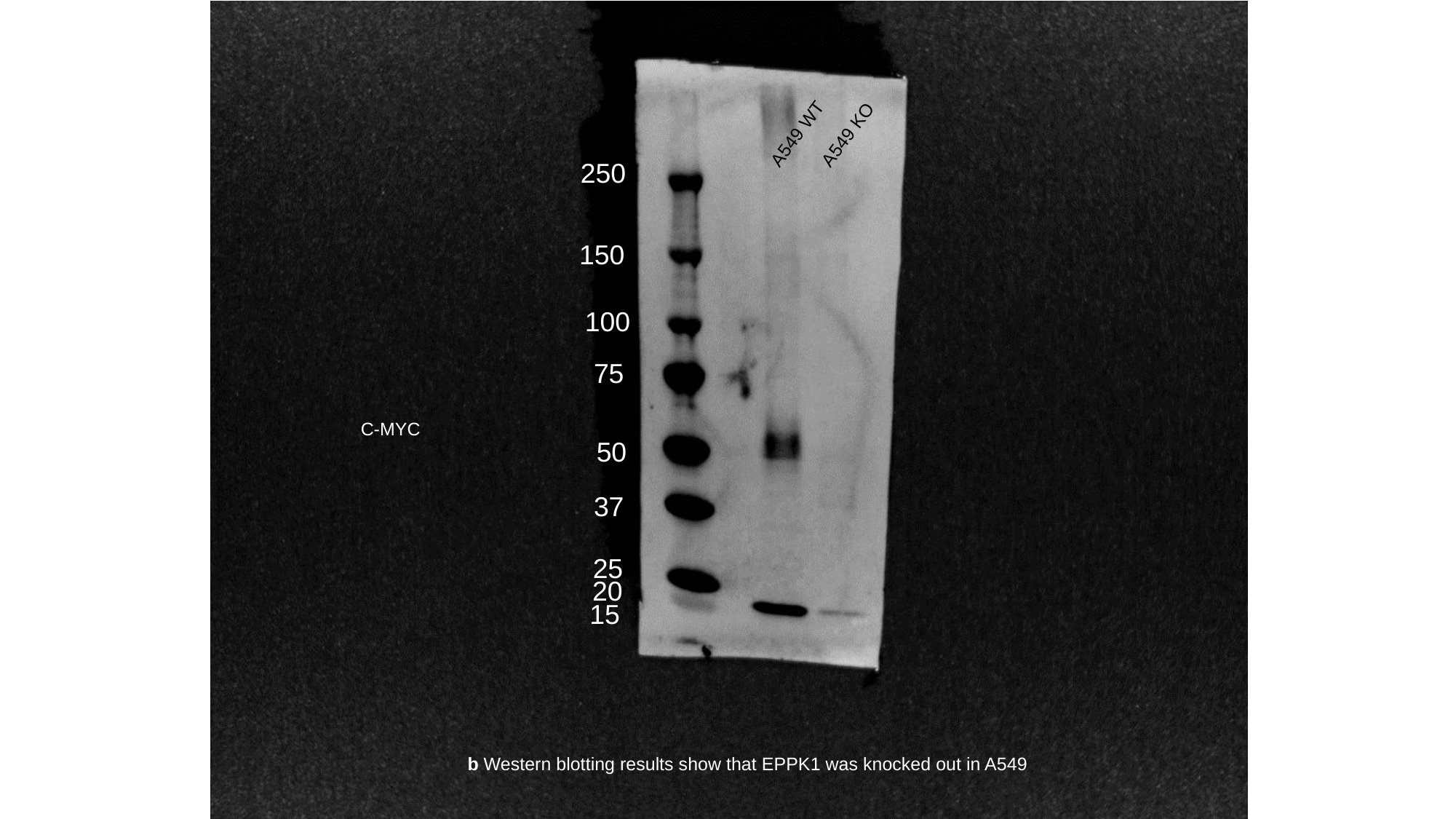

A549 WT
A549 KO
250
150
100
75
C-MYC
50
37
25
20
15
b Western blotting results show that EPPK1 was knocked out in A549

## Slide 17
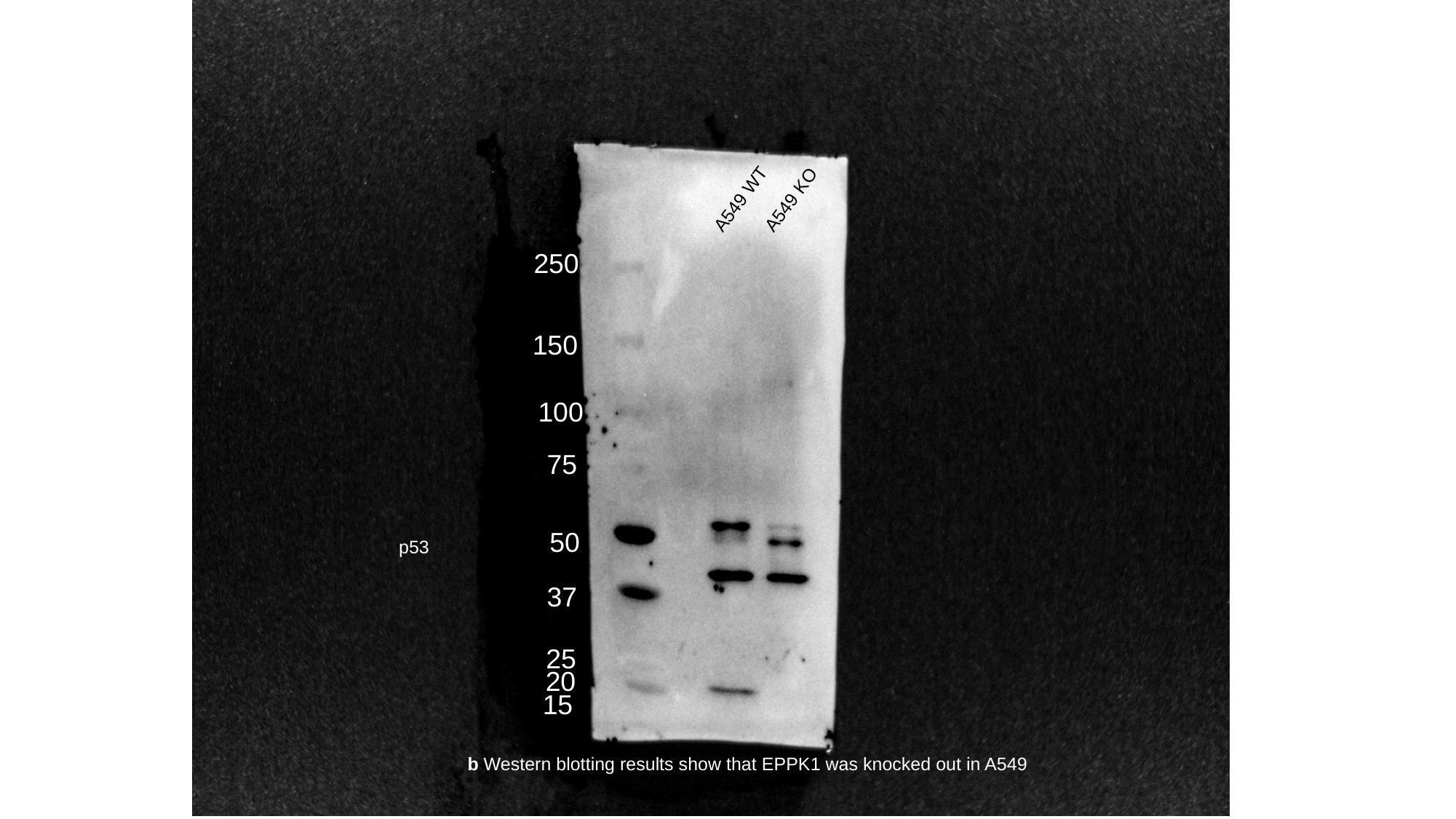

A549 WT
A549 KO
250
150
100
75
50
p53
37
25
20
15
b Western blotting results show that EPPK1 was knocked out in A549

## Slide 18
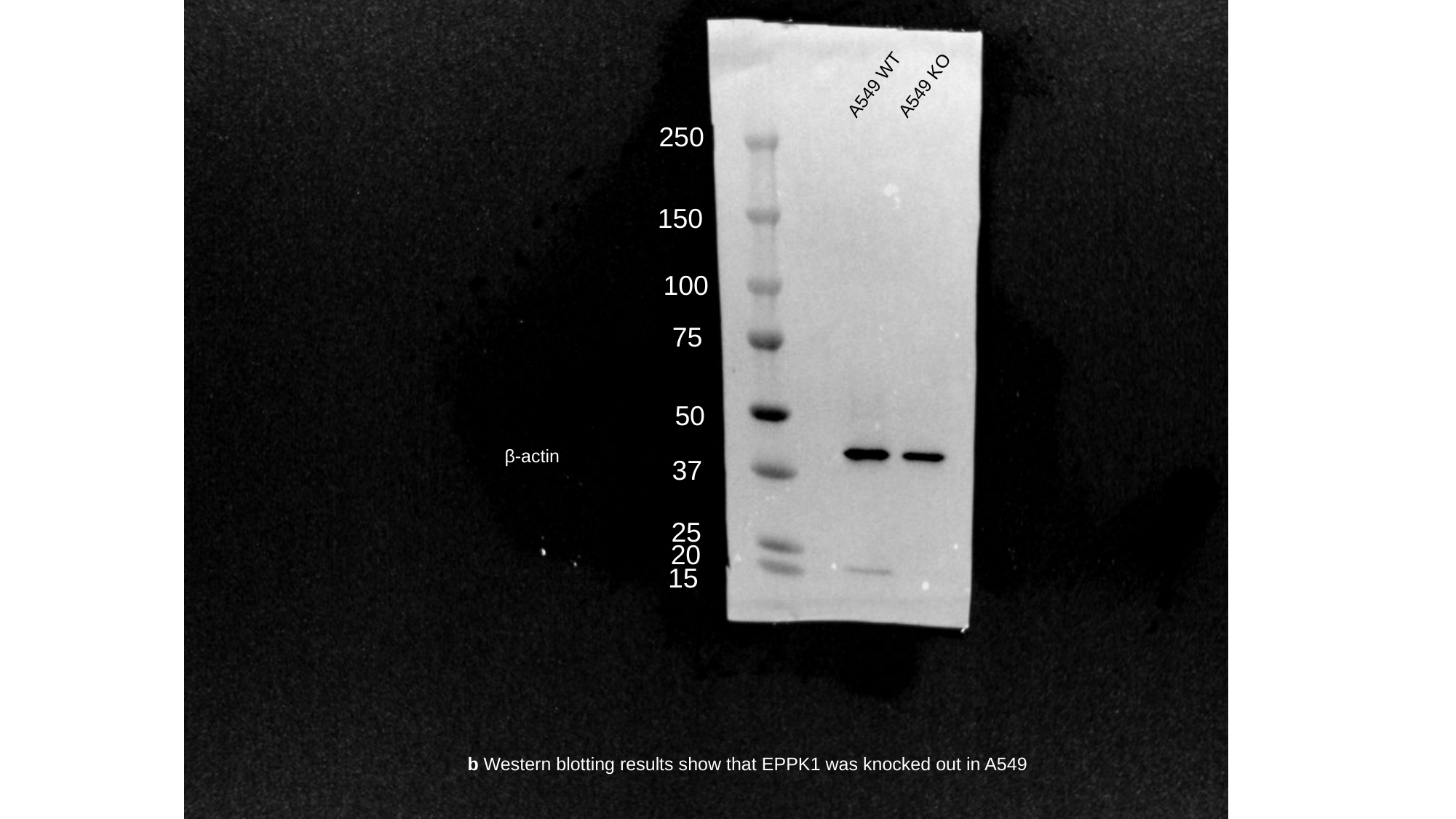

A549 WT
A549 KO
250
150
100
75
50
β-actin
37
25
20
15
b Western blotting results show that EPPK1 was knocked out in A549
